# Elevated systemic inflammatory responses, factors associated with physical and mental quality of life, and prognosis of hepatocellular carcinoma

**DOI:** 10.18632/aging.102889

**Published:** 2020-03-07

**Authors:** Yang Deng, Jian Zhu, Ziyi Liu, Maosheng Huang, David W. Chang, Jian Gu

**Affiliations:** 1 Department of Surgery, Ruijin Hospital, Shanghai Jiao Tong University School of Medicine, Shanghai 200025, China; 2 Department of Epidemiology, The University of Texas MD Anderson Cancer Center, Houston, TX 77030, USA

**Keywords:** hepatocellular carcinoma, quality of life, age at diagnosis, systemic inflammatory response, Short Form-12

## Abstract

Impaired quality of life (QOL) is common in hepatocellular carcinoma (HCC) patients. In this study, we used a large hospital-based multiethnic HCC patient cohort to systematically identify factors associated with QOL and investigate the prognostic value of QOL.

The Short Form-12 questionnaire was used to assess QOL. The Physical Component Summary (PCS) and Mental Component Summary (MCS) scores were categorized into three groups (low, medium, and high) and ordered logistic regression analysis was used to analyze the association of PCS and MCS scores with patient characteristics. The association of PCS and MCS scores with mortality was assessed by Cox regression analysis.

Notably, a panel of elevated systemic inflammatory response markers was associated with poor QOL. Other significant factors associated with QOL included age, liver function, sex, smoking, HCC etiology, and major clinical features. Patients with low (hazard ratio [95% CI], 1.72 [1.36-2.17]) and medium (1.52 [1.23-1.89]) PCS scores exhibited higher risks of death compared to patients with high PCS score. The association of MCS with the risk of death was not significant. These observations were consistent across all the different ethnicities.

The identified factors associated with QOL may help clinicians formulate interventions to improve QOL and outcomes in HCC patients.

## INTRODUCTION

Liver cancer was the sixth most common cancer and the fourth leading cause of cancer mortality globally with an estimated 841,080 new cases and 781,631 deaths in 2018 [1]. In the United States, the age-adjusted incidence rates of liver cancer tripled between 1975 and 2011 because of the increased burden of hepatitis C infection [2]. The 5-year relative survival rate is approximately 18% for all stages combined and only 3% for distant stages [3]. Hepatocellular carcinoma (HCC) accounts for 75% to 85% of primary liver cancers [1] and is a leading cause of death among patients diagnosed with cirrhosis [4].

Quality of life (QOL) has become a subject of paramount importance for liver cancer patients [5, 6]. The development of HCC is closely associated with an established background of chronic liver disease and impaired QOL [7, 8]. Previous studies have consistently shown that QOL is a prognostic indicator of survival in patients with HCC, with a high baseline QOL being associated with longer overall survival (OS) [9–15].

Previous studies found that some demographic, psychological, and clinical factors play an important role in determining the QOL of HCC patients [7, 13, 16–19]. However, these studies have been limited by small sample sizes (36 to 538 patients). In addition, most studies have focused on advanced HCC without considering early-stage HCC [9–11, 19]. Although the prognostic value of QOL has been demonstrated in Chinese [10, 12] and Caucasian patients with HCC [9, 11, 13, 14], no study has evaluated the prognostic value and the factors associated with QOL in HCC patients across different races and ethnicities. Therefore, in this study, we addressed these issues in a large cohort of HCC patients that was racially and ethnically diverse and encompassed all stages and different etiologies [15].

## RESULTS

### Patient characteristics

A total of 735 patients were recruited in this study. The characteristics of the HCC patients and the distributions of PCS and MCS by patient characteristics in this study are shown in Supplementary Table 1. The mean age was 61.7 years (standard deviation [SD]: 12.0 years); the patients were mostly men (549 [74.7%]) and non-Hispanic white (469 [63.8%]). The most common etiologies were HCV and/or HBV infection (186 [25.3%]) and alcohol abuse (186 [25.3%]). Five hundred eighty-four (79.5%) patients were diagnosed with Child-Pugh A, and 516 (70.6%) were diagnosed with stages III and IV. Among the 192 patients who had undergone prior treatment, 67 (34.9%) had been treated by curative therapy (surgical and ablation therapies). The mean PCS and MCS scores were 37.9 (SD: 12.1) and 46.3 (SD: 11.3), respectively. A number of variables were associated with PCS and MCS scores, for example, current smokers had significantly lower PCS and MCS scores than never smokers and patients with worse clinical features and symptoms (e.g., worse Child-Pugh scores, presence of cirrhosis or portal vein thrombosis, higher tumor stage, and increased AFP, CA19-9, ALP, bilirubin, or serum albumin) had lower PCS and MCS scores. Notably, a panel of elevated systemic inflammatory response (SIR) markers was associated with lower PCS and MCS scores. Patients with high white blood cell (WBC) counts, high neutrophil counts, and high neutrophils-to-lymphocyte ratio (NLR) had lower PCS and MCS scores, whereas patients with high lymphocytes and high lymphocytes-to-monocyte ratio (LMR) had significantly higher PCS and MCS scores (Supplementary Table 1).

### Risk factors for low PCS and MCS scores

We then performed ordered logistic regression analysis to analyze the associations of patient characteristics with PCS and MCS scores. In univariate analysis (Supplementary Table 2), 25 factors were significantly associated with PCS, 15 of which remained significant in multivariate analysis (smoking, Child-Pugh score, portal vein thrombosis, tumor stage, comorbidity, prior treatment, CA199, ALP, serum albumin, WBC, lymphocytes, monocytes, neutrophils, NLR, LMR). One factor (sex) was significant on multivariate analysis but not on univariate analysis. Seventeen factors were associated with MCS in univariate analyses, among which 10 factors (age at diagnosis, sex, etiology, tumor stage, directed bilirubin, serum albumin, WBC, neutrophils, NLR, LMR) remained significant in multivariate analysis. One factor (comorbidity) was significant on multivariate analysis but not on univariate analysis. In multivariate logistic regression analyses (Supplementary Table 2), patients with abnormal WBC counts (> 11 × 10^9^/L) were 3.19-fold more likely to have lower PCS scores (95% CI, 1.46-6.97, *P* = 0.004) and 2.72-fold more likely to have lower MCS scores (95% CI, 1.20-6.15, *P* = 0.02) than were patients with normal WBC counts (4-11 × 10^9^/L). Similar results were found for neutrophil cell count (OR_PCS_: 2.67 [1.38-5.17], *P* = 0.004; OR_MCS;_ 2.57 [1.31-5.07], *P* = 0.006). Patients with an NLR > 4.0 were more likely to have lower PCS scores (2.14 [1.30-3.53]; *P* = 0.003) and lower MCS scores (1.88 [1.14-3.12]; *P* = 0.01) than were patients with an NLR ≤ 4.0. Patients with serum albumin level < 3.5 g/dl (2.11 [1.12-3.98]; *P* = 0.02) and < 3.2 g/dl (5.83 [2.38-14.28]; *P* < 0.001) were more likely to have lower PCS scores than were patients with serum albumin level ≥ 3.5 g/dl (*P*-trend < 0.001). Patients with serum albumin level < 3.2 g/dl were more likely to have lower MCS scores (2.75 [1.16-6.48]; *P* = 0.02) than were patients with serum albumin level ≥ 3.5 g/dl.

Age at diagnosis, etiology, and direct bilirubin were significantly associated with MCS. Patients aged ≥ 75 years were less likely to have lower MCS scores than were patients aged < 55 years (0.53 [0.31-0.90]; *P* = 0.02). Patients with direct bilirubin > 0.4 mg/dl were more likely to have lower MCS scores than were patients with direct bilirubin ≤ 0.4 mg/dl (2.23 [1.15-4.32]; *P* = 0.02).

Smoking status, Child-Pugh score, portal vein thrombosis, prior treatment, CA19-9, alkaline phosphatase, lymphocyte and monocyte counts, and LMR were significantly associated with PCS. Patients with Child-Pugh classification B (1.97 [1.33-2.92]; *P* < 0.001) and C (3.57 [1.31-9.73]; *P* = 0.01) were more likely to have lower PCS scores than were those with Child-Pugh classification A (*P*-trend < 0.001). Patients with alkaline phosphatase > 200 U/L (2.70 [1.49-4.89]; *P* = 0.001) were more likely to have lower PCS scores than were patients with normal alkaline phosphatase (≤ 126 U/L). Patients with an LMR > 2.9 were more likely to have higher PCS scores (0.53 [0.33-0.86]; *P* = 0.009) than were patients with an LMR ≤ 2.9. African Americans were more likely to have lower PCS scores than were non-Hispanic whites, which was borderline statistically significant on multivariate analysis (1.63 [0.94-2.84]; *P* = 0.08). Similar factors associated with poorer PCS and MCS were found for different races and ethnicities (Supplementary Tables 3 and 4).

### Association between PCS and MCS scores and survival

After a median follow-up time of 76.9 months (95% CI: 63.6-93.9 months), 560 (76.2%) patients had died. The median survival time (MST) for all patients was 14.4 months (95% CI: 12.6-16.3 months). The 1-year and 5-year relative OS rates were 56.3% and 14.7%, respectively.

**Figure 1:**
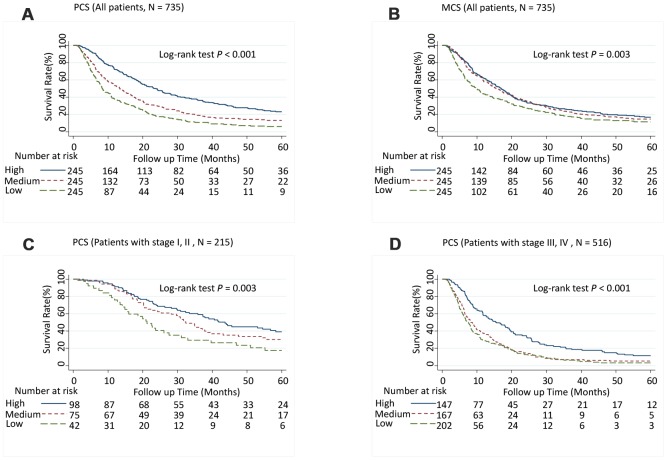
**Five-year overall survival rates of hepatocellular carcinoma patients by Physical Component Summary (PCS) and Mental Component Summary (MCS) scores, categorized into tertiles.** (**A**) PCS (Overall population, N = 735), (**B**) MCS (Overall population, N = 735), (**C**) PCS (Patients with stages I and II, N = 215), and (**D**) PCS (Patients with stages III and IV, N = 516). Higher scores indicate a better physical or mental quality of life. PCS: High, ≥ 45.0; Medium, ≥ 30.5, < 45.0; Low, < 30.5. MCS: High, ≥ 54.4; Medium, ≥ 41.3, < 54.4; Low, < 41.3.

**Figure 2:**
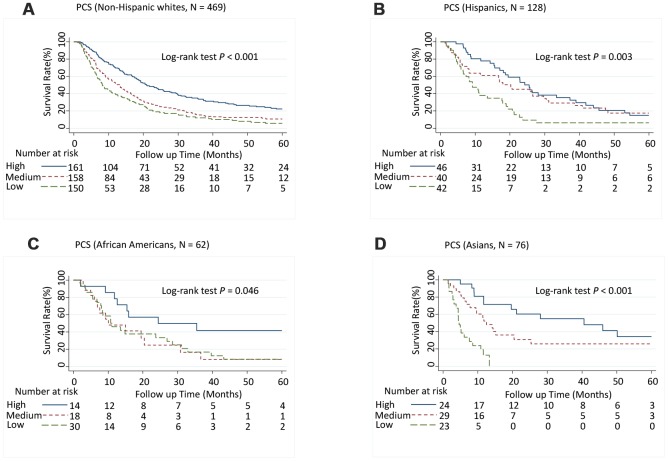
**Five-year overall survival rates of hepatocellular carcinoma patients by Physical Component Summary (PCS) scores, stratified by race and ethnicity.** (**A**) Non-Hispanic white (N = 469), (**B**) Hispanic (N = 128), (**C**) African American (N = 62), and (**D**) Asian (N = 76). PCS scores were categorized into tertiles. Higher scores indicate a better physical quality of life. PCS: High, ≥ 45.0; Medium, ≥ 30.5, < 45.0; Low, < 30.5.

Patients with medium PCS (hazard ratio [95% CI], 1.52 [1.23-1.89]; *P* < 0.001) and low PCS scores (1.72 [1.36-2.17]; *P* < 0.001) had significantly increased risk of death (*P*-trend < 0.001) (Table 1) and significantly shorter MST than did patients with high PCS scores (log-rank *P* < 0.001; Figure 1A). Although patients with low MCS scores had a significantly shorter MST than did those with high MCS group scores (log-rank *P* = 0.003; Figure 1B), the association between MCS score and the risk of death was not significant after adjusting for confounders in multivariate Cox analysis (Table 1). When further stratified by stage or race and ethnicity, the effect of low PCS score was consistent between patient with stage I and II disease and stage III and IV disease (Figure 1C and 1D) and among all races and ethnicities (Figure 2). We also performed stratified analyses by sex, prior treatment, cirrhosis, and portal vein hypertension history and found similar results (Supplementary Table 5 and Supplementary Figures 1, 2).

**Table 1:** Association of PCS/MCS score with five-year overall survival.

**Variables**	**PCS ^a^**					**MCS ^b^**			
	**Adjusted HR ^c^ (95% CI)**	***P* value**	**MST**	**Log rank *P***		**Adjusted HR ^c^ (95% CI)**	***P* value**	**MST**	**Log rank *P***
**All patients**									
High score	1.00 (Ref)		23.8			1.00 (Ref)		17.0	
Medium score	1.52 (1.23-1.89)	**< 0.001**	13.4			0.95 (0.77-1.17)	0.61	15.8	
Low score	1.72 (1.36-2.17)	**< 0.001**	8.4	**< 0.001**		1.12 (0.91-1.39)	0.29	10.0	**0.003**
***P* for trend**		**< 0.001**					0.30		
**Non-Hispanic white**									
High score	1.00 (Ref)		20.9			1.00 (Ref)		15.5	
Medium score	1.53 (1.16-2.00)	**0.002**	13.2			0.91 (0.69-1.19)	0.49	15.0	
Low score	1.52 (1.13-2.05)	**0.006**	8.2	**< 0.001**		1.13 (0.85-1.49)	0.41	10.0	0.09
***P* for trend**		**0.005**					0.43		
**Hispanic**									
High score	1.00 (Ref)		25.5			1.00 (Ref)		22.8	
Medium score	1.22 (0.71-2.10)	0.46	20.3			1.01 (0.60-1.72)	0.96	20.6	
Low score	2.83 (1.58-5.05)	**< 0.001**	9.20	**0.003**		1.99 (1.18-3.37)	**0.01**	9.2	0.09
***P* for trend**		**< 0.001**					**0.01**		
**African-American**									
High score	1.00 (Ref)		24.4			1.00 (Ref)		15.0	
Medium score	13.89 (2.77-69.73)	**0.001**	10.1			1.75 (0.55-5.56)	0.34	15.4	
Low score	7.84 (1.41-43.74)	**0.02**	11.1	**0.046**		0.39 (0.10-1.57)	0.19	10.1	0.72
***P* for trend**		**0.03**					0.17		
**Asian**									
High score	1.00 (Ref)		40.6			1.00 (Ref)		10.9	
Medium score	0.99 (0.36-2.74)	0.99	12.5			2.97 (0.91-9.67)	0.07	11.7	
Low score	8.45 (2.29-31.16)	**0.001**	4.6	**< 0.001**		5.48 (1.52-19.81)	**0.009**	9.6	0.18
***P* for trend**		**0.003**					**0.008**		
**Stage (I and II)**									
High score	1.00 (Ref)		43.7			1.00 (Ref)		37.9	
Medium score	1.44 (0.94-2.19)	0.09	32.4			1.21 (0.77-1.89)	0.41	29.7	
Low score	2.66 (1.56-4.53)	**< 0.001**	21.2	**0.003**		0.93 (0.55-1.57)	0.78	34.9	0.86
***P* for trend**		**< 0.001**					0.87		
**Stage (III and IV)**									
High score	1.00 (Ref)		15.6			1.00 (Ref)		10.8	
Medium score	1.69 (1.30-2.21)	**< 0.001**	8.3			0.89 (0.69-1.15)	0.37	10.0	
Low score	1.59 (1.21-2.09)	**< 0.001**	7.6	**< 0.001**		1.21 (0.94-1.55)	0.14	7.3	**0.02**
***P* for trend**		**< 0.001**					0.13		

Abbreviations: CI, confidence interval; HR, hazard ratio; MCS, Mental Component Summary; MST, median survival time; PCS, Physical Component Summary.

^a^ PCS: High, ≥ 45.0; Medium, ≥ 30.5, < 45.0; Low, < 30.5.

^b^ MCS: High, ≥ 54.4; Medium, ≥ 41.3, < 54.4; Low, < 41.3.

^c^ Adjusted for sex, age at diagnosis, race, BMI, Child-Pugh score, cirrhosis, portal hypertension, portal vein thrombosis, cancer stage, histologic grade, comorbidity, and prior treatment.

### Meta-analyses of prognostic values of PCS and MCS

**Figure 3:**
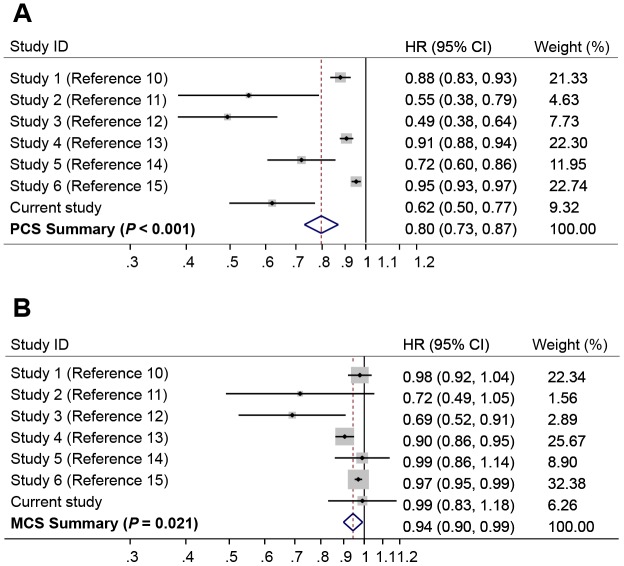
Forest plots of meta-analyses for the associations of risk of death with (**A**) Physical Component Summary (PCS) scores, and (**B**) Mental Component Summary (MCS) scores. The summary HR was estimated using random effects model.

A number of publications have evaluated the associations of PCS and MCS with overall survival in HCC patients [10–15]. We performed a random effect meta-analysis for the associations of HCC survival with PCS or MCS. The results showed that higher PCS was strongly associated with a reduced risk of death (HR = 0.80, 95% CI = 0.73-0.87, *P* < 0.001) (Figure 3A), whereas higher MCS was associated with a modestly reduced risk of death (HR = 0.94, 95% CI, 0.90-0.99, *P* = 0.021) (Figure 3B). There was significant heterogeneity between different studies for the association of survival with PCS (I^2^ = 90.4%, *P* < 0.001) and MCS (I^2^ = 61.0%, *P* = 0.017).

## DISCUSSION

This is the first prospective study using the Short Form-12 version 1 (SF-12v1) questionnaire to explore associated factors and prognostic value of QOL in a large cohort of racially and ethnically diverse patients with HCC. We identified multiple socio-demographic, clinical, and biochemical factors were associated with QOL; these factors were similar among different races and ethnicities. More importantly, we found that physical QOL after diagnosis was a significant prognostic indicator for survival and this effect was consistent across different races and ethnicities.

A notable finding of this study was the association between inflammatory response markers and QOL. To the best of our knowledge, this is the first study to demonstrate that elevated SIR is related to poor QOL in HCC patients. Elevated SIR markers, including elevated WBC, neutrophil, and monocyte counts, a high NLR, a decreased lymphocyte count, and a low LMR, have all been shown to be associated with clinical outcomes in advanced cancer patients [20]. One recent study also showed that elevated SIR was independently associated with deterioration in QOL parameters in patients with various advanced cancers [21]. However, in that study, HCC was not specified. The underlying mechanism of the association of SIR and QOL is unclear. A previous study reported an association between systemic inflammation and the presence of symptoms such as pain, anorexia, and fatigue in patients with advanced cancer [22]. There is growing evidence suggesting that host SIR drives both disease progression and the symptoms that lead to poorer QOL [23]. A significant association has also been reported between SIR and self-reported emotional and social function in patients with advanced cancer [22]. It has been recognized that SIR has an impact on both the central nervous system and brain functions, including cognition, mood, and other psychological symptoms [24], which influences the mental QOL. The SIR in cancer patients could cause profound changes at the genomic, intracellular, cellular and systemic levels [25]. A key pathway connecting these changes at multiple levels is the interleukin-6/Janus kinase/signal transducer and activator of transcription (IL-6/JAK/STAT) pathway [26]. Chronic activation of the IL-6/JAK/STAT pathway in the tumor and its microenvironment produces a deregulated inflammatory cascade at cellular and systemic levels (increased C-reactive protein, neutrophil counts and decreased albumin) [20]. Given the association of SIR with QOL and survival, targeting IL-6/JAK/STAT pathway to attenuate systemic inflammation should be helpful in improving QOL and survival in HCC patients.

Aging is associated with decreasing QOL for all functioning scales in general populations [27]. Previous studies have shown that elderly patients with HCC had poor physical QOL [7, 18, 28]. One recent study found that social functioning and financial problems improve with age, while physical functioning deteriorates with age in cancer patients [29]. Interestingly, our study showed that patient aged ≥ 75 years had better mental QOL, which was consistent with a previous study showing that younger survivors reporting more unmet needs in emotional/mental health domains [30]. One recent study also reported that older survivors of colon and rectal cancer had higher functioning and lower symptom burden compared with those diagnosed at < 50 years of age [31]. This may be because older adults have more experience in coping with severe illness [32], attend fewer social activities, and bear less financial burden [29]. Personalized education programs and appropriate supportive interventions should be formulated for younger patients with HCC.

Our study showed that women, African Americans, current smoker, and patients with comorbidities were more likely to have a poor physical and/or mental QOL. Several studies have shown that female sex is associated with poor QOL in HCC patients [7, 13, 16, 28]. One possible reason is that female HCC patients are more likely to be stigmatized since HCC is often considered to be associated with alcoholism or drug use [13]. The second possible reason is that the somatic symptoms influence QOL more deleteriously among women than among men [33]. Previous studies reported that current smokers with cancer had a poor QOL [34, 35], possibly due to airway inflammation, decreased lung function, reduced mucociliary clearance, and more severe pain sensations [36, 37]. Consistent with previous reports [38, 39], we also found that African Americans had poorer physical QOL than did non-Hispanic whites. The main reason for this disparity is likely the low socio-economic status (SES) of African Americans [40]. Low SES has a significant impact on access to medical care and is associated with later disease stage at diagnosis and higher rates of comorbidities in minority populations [41, 42].

The deterioration of liver disease and liver function could strongly influence QOL [13]. As expected, we found that the severity of disease and poor liver function were associated with poor QOL in HCC patients. Previous studies also reported that patients with high tumor stage [28, 43], poor Child-Pugh scores [13, 16, 18], and poor liver function [7, 19] had poor QOL.

Physical QOL has been consistently associated with survival in HCC using different QOL assessment instruments [10–14]. A recent study also showed that preoperative physical QOL score was predictive of OS after surgical resection of HCC [15]. Poor physical QOL may decrease patients’ treatment compliance, resulting in premature termination of treatment. In addition, physical QOL could influence therapeutic decision making in HCC patients [5]. Interestingly, we found that mental QOL had no significant prognostic value. This finding is consistent with previous reports that emotional functioning and social well-being are not significantly associated with survival after adjusting for socio-demographic and clinical variables [10–13]. Our findings provide strong evidence to support the prognostic value of physical QOL for HCC in this racially and ethnically diverse patient population; QOL measures may help clinicians predict the prognosis of HCC patients and should be considered a complementary prognostic tool in clinical practice.

The major strength of this study is its large, diverse HCC patient population that allowed us to generalize our findings to different racial and ethnic groups. In addition, markers of SIR were included in the analysis, which allowed us to assess the relationship between SIR and QOL. Our study has a few limitations. First, we did not perform a longitudinal assessment of QOL; thus, we could not determine whether changes in QOL were predictive of survival. Second, although this study had a large patient cohort, the number of patients with available laboratory data was relatively small, which may have reduced the statistical power in a few stratified analyses. Third, we did not collect diet and physical activity information, which may be associated with PCS and MCS scores. Finally, although most of our findings with regard to the risk factors associated with QOL and the prognostic value of QOL were consistent with literature, our panel of inflammatory markers as risk factors for QOL warrants validation in independent external HCC patient cohorts.

In conclusion, we found that physical QOL after diagnosis was an independent prognostic indicator for HCC. QOL measurements may help clinicians identify subpopulations of HCC patients who are at high risk of poor survival, which may be helpful in monitoring patients and formulating interventions. We also identified multiple factors associated with QOL in HCC patients. These factors could help clinicians tailor individualized interventions to improve QOL and survival in HCC patients. Further studies are necessary to develop a QOL-integrated prognostic model that can increase the accuracy of mortality prediction in HCC patients.

## MATERIALS AND METHODS

### Patients

The participants were patients with HCC who had been diagnosed between October 1999 and April 2012, and who had been included in The MD Anderson Cancer Patients and Survivors Cohort Study (MDA-CPSC) [44], a prospective hospital-based cohort study conducted in the United States. At their initial visit, all participants completed a patient history form that collected epidemiologic, socio-demographic, and risk factor information. The patient history form also assessed QOL using the generic, validated SF-12v1 questionnaire [45]. Clinical information was abstracted from the institutional Tumor Registry after patients were enrolled and during treatment. Laboratory tests, including tests of bilirubin, albumin, and prothrombin activity, were performed to evaluate liver function. The WBC count, NLR, and LMR from peripheral blood sample were used to evaluate SIR. Lab tests were performed at the time of diagnosis or during treatment. If multiple lab tests were done, the test results obtained closest to the time of QOL assessment were selected. The American Joint Committee on Cancer 7^th^ edition of the TNM staging system was used for tumor staging [46]. This study was approved by the institutional review board at The University of Texas MD Anderson Cancer Center (Houston, Texas), and all participants provided the informed consent.

### Eligibility and exclusion criteria

Only HCC patients who had completed the patient history form and SF-12v1 questionnaire within one year of diagnosis were included in this study. Patients aged < 18 years, with multiple primary tumors and cognitive impairment, were excluded. The final number of patients recruited in this study was 735. The diagnosis of HCC was confirmed by either histological examination, a combination of radiological and biochemical findings (a-fetoprotein ≥ 400 ng/mL), or two typical radiological findings (ultrasonography, triphasic dynamic computed tomography, or magnetic resonance imaging) [47].

### SF-12v1 questionnaire

The SF-12v1 questionnaire is a 12-item generic measure of health status that evolved from the Short Form-36 questionnaire. This multipurpose questionnaire yields scores for eight domains: physical functioning, role-physical, bodily pain, general health, vitality, social functioning, role-emotional, and mental health. The eight domains of this questionnaire can be summarized into two indices: the PCS and MCS. After reversal and recalibration, the scores can be transformed to a 0-100 scale and then to a norm-based score, with higher scores representing a higher/healthier level of QOL [45].

### Statistical analysis

The PCS (high: ≥ 45.0; medium: ≥ 30.5, < 45.0; low: < 30.5) and MCS (high: ≥ 54.4; medium: ≥ 41.3, < 54.4; low: < 41.3) scores were categorized into tertiles based on their distribution in the patient population. The difference in mean PCS and MCS scores between categories of host characteristics was analyzed by Wilcoxon Rank Sum Test. Ordered logistic regression analysis was used to estimate the associations between patient characteristics and categorical PCS and MCS scores. We independently assessed all variables using a univariate model. Variables that were significant on univariate analysis were included in a multivariate model, and forward selection was used to eliminate variables with a *P* value > 0.05. Survival time was defined as the period from diagnosis to death or last follow-up and five-year OS was analyzed. Multivariate Cox proportional hazards models were used to analyze the associations of PCS and MCS scores with the risk of death adjusting for potential confounders (sex, age at diagnosis, race, BMI, Child-Pugh score, cirrhosis, portal hypertension, portal vein thrombosis, cancer stage, histological grade, comorbidity, and prior treatment). These confounders were selected using a stepwise model building procedure based on a significant level of < 0.05. We performed sensitivity analyses by adjusting different sets of confounders in several different models and the risk estimates were similar. No single confounder had a dramatic effect on risk estimate. We also tested the correlation among the variables using Spearman’s rank correlation and only included independent variables in multivariate logistic regression and Cox analysis. MST for the high, medium, and low PCS and MCS groups were determined using the Kaplan-Meier curve and compared using the log-rank test. We performed a random-effect model meta-analysis for the associations of PCS and MCS scores with the risk of death and quantified between-studies heterogeneity with I^2^ (I^2^ < 50% indicates no heterogeneity). All statistical tests were two-sided, and *P* values < 0.05 were considered statistically significant. Statistical analyses were conducted using Stata software version 14.2 (StataCorp LP, College Station, Texas).

## Supplementary Material

Supplementary Figures

Supplementary Table 1

Supplementary Table 2

Supplementary Table 3

Supplementary Table 4

Supplementary Table 5
