# Supplementary material for: Elevated systemic inflammatory responses, factors associated with physical and mental quality of life, and prognosis of hepatocellular carcinoma
Source: Aging (Albany NY). 2020 Mar 7;12(5):4357–70. doi: 10.18632/aging.102889 (PMC7093167; doi:10.18632/aging.102889)
Supplement: Supplementary Figures [file aging-12-102889-s006..pdf]

# SUPPLEMENTARY FIGURES

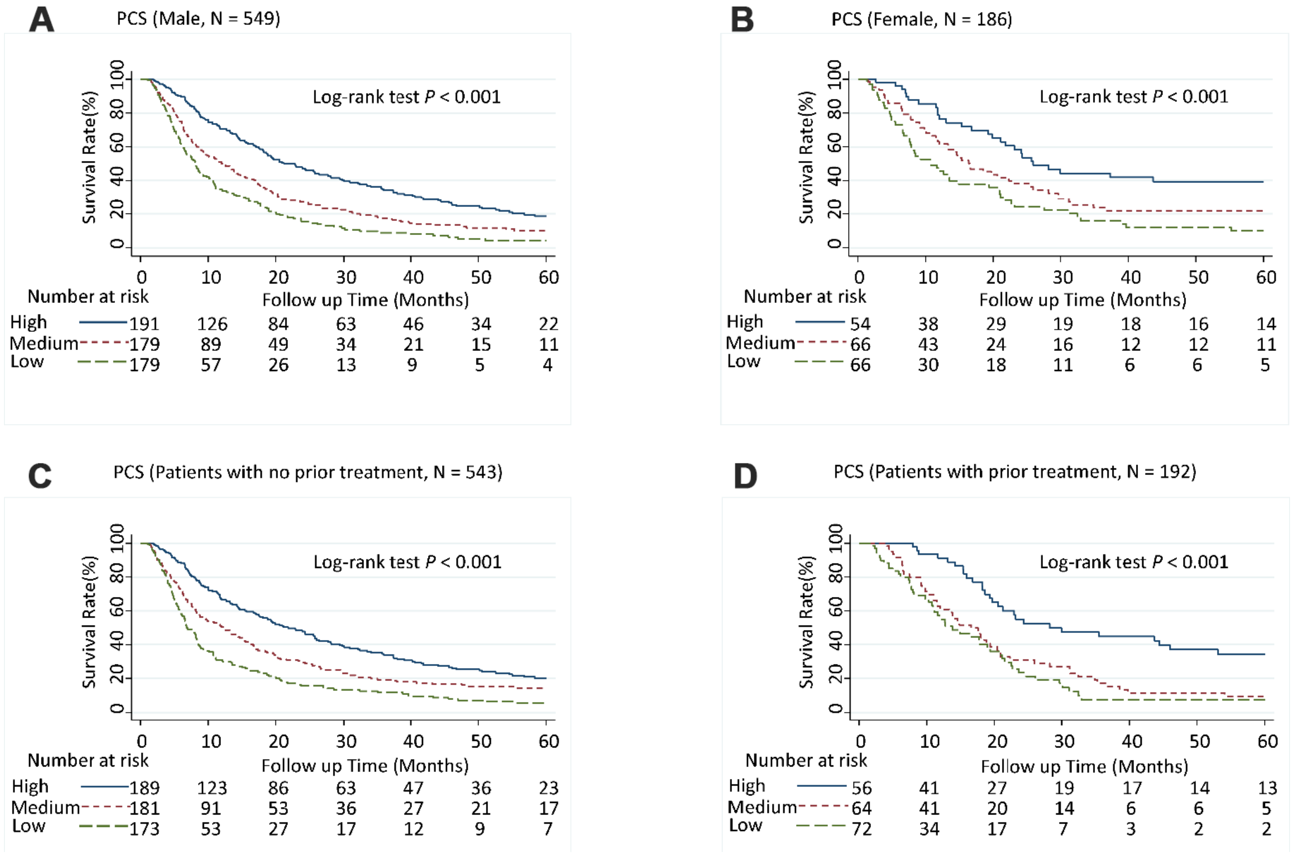


**Supplementary Figure 1. Five-year overall survival rates of hepatocellular carcinoma patients by Physical Component Summary (PCS) scores, stratified by sex and prior treatment history.** (**A**) Male (N = 549), (**B**) Female (N = 186), (**C**) Patients with no prior treatment (N = 543), and (**D**) Patients with prior treatment (N = 192). PCS scores were categorized into tertiles; higher scores indicate a better physical quality of life. PCS: High, ≥ 45.0; Medium, ≥ 30.5, < 45.0; Low, < 30.5.

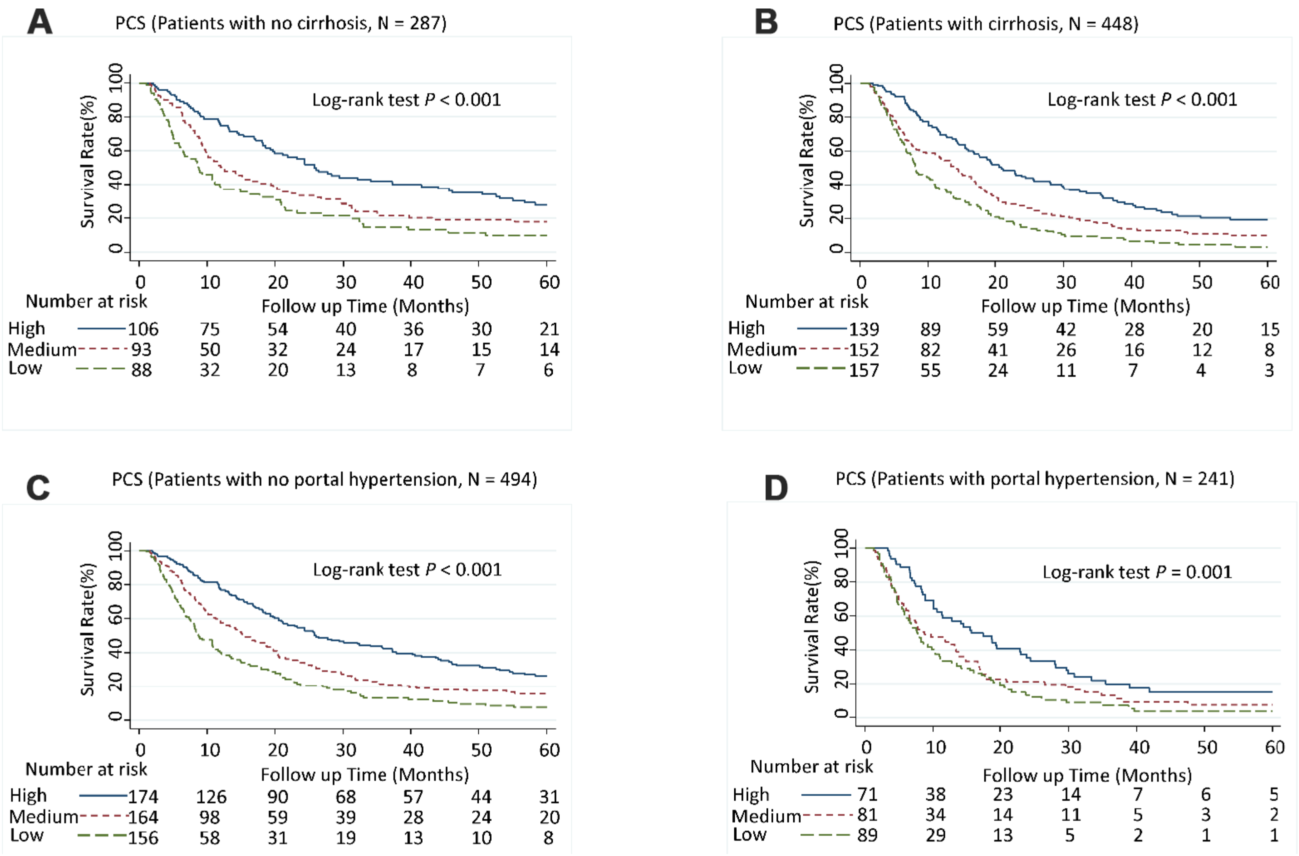


**Supplementary Figure 2. Five-year overall survival rates of hepatocellular carcinoma patients by Physical Component Summary (PCS) scores, stratified by cirrhosis and portal hypertension history.** (**A**) Patients with no cirrhosis (N = 287), (**B**) Patients with cirrhosis (N = 448), (**C**) Patients with no portal hypertension (N = 494), (**D**) Patients with portal hypertension (N = 241). PCS scores were categorized into tertiles; higher scores indicate a better physical quality of life. PCS: High, ≥ 45.0; Medium, ≥ 30.5, < 45.0; Low, < 30.5.
