# Supplementary material for: Elevated systemic inflammatory responses, factors associated with physical and mental quality of life, and prognosis of hepatocellular carcinoma
Source: Aging (Albany NY). 2020 Mar 7;12(5):4357–70. doi: 10.18632/aging.102889 (PMC7093167; doi:10.18632/aging.102889)
Supplement: Supplementary Table 1 [file aging-12-102889-s003..docx]

**Supplementary Table 1. Distribution of PCS and MCS by selected patient characteristics**

| **Characteristic** | **N (%) ^a^** |  | **PCS** |  |  | **MCS** |  |
| --- | --- | --- | --- | --- | --- | --- | --- |
|  |  |  | **Mean (SD)** | ***P* value** |  | **Mean (SD)** | ***P* value** |
| **Total** |  |  | 37.9 (12.1) |  |  | 46.3 (11.3) |  |
| **Age at diagnosis, years** |  |  |  |  |  |  |  |
| < 55 | 182 (24.8) |  | 36.7 (12.3) |  |  | 45.1 (10.6) |  |
| ≥ 55, < 65 | 237 (32.2) |  | 36.8 (12.2) | 0.84 |  | 45.8 (11.2) | 0.50 |
| ≥ 65, < 75 | 206 (28.0) |  | 39.6 (12.0) | **0.03** |  | 46.5 (12.0) | 0.12 |
| ≥ 75 | 110 (15.0) |  | 39.1 (11.1) | 0.07 |  | 48.9 (11.0) | **0.002** |
| ***P* for trend** |  |  |  | **0.01** |  |  | **0.002** |
| **Sex** |  |  |  |  |  |  |  |
| Male | 549 (74.7) |  | 38.1 (12.1) |  |  | 46.7 (11.4) |  |
| Female | 186 (25.3) |  | 37.3 (12.0) | 0.48 |  | 45.1 (11.1) | 0.07 |
| **Race/ethnicity** |  |  |  |  |  |  |  |
| Non-Hispanic white | 469 (63.8) |  | 38.1 (12.0) |  |  | 46.6 (11.2) |  |
| Hispanic | 128 (17.4) |  | 38.6 (12.1) | 0.70 |  | 46.5 (11.6) | 0.99 |
| African American | 62 ( 8.4) |  | 35.2 (12.5) | 0.06 |  | 44.3 (11.7) | 0.13 |
| Asian | 76 (10.3) |  | 37.8 (12.1) | 0.95 |  | 46.0 (11.7) | 0.57 |
| **Alcohol consumption** |  |  |  |  |  |  |  |
| Never | 292 (39.7) |  | 37.6 (11.7) |  |  | 46.6 (10.8) |  |
| Former | 330 (44.9) |  | 37.3 (12.1) | 0.74 |  | 45.6 (11.6) | 0.34 |
| Current | 113 (15.4) |  | 40.5 (12.7) | **0.03** |  | 47.5 (11.8) | 0.30 |
| ***P* for trend** |  |  |  | 0.11 |  |  | 0.63 |
| **Smoking status** |  |  |  |  |  |  |  |
| Never | 248 (33.7) |  | 38.6 (11.6) |  |  | 46.8 (10.8) |  |
| Former | 377 (51.3) |  | 38.4 (12.3) | 0.71 |  | 47.1 (11.5) | 0.55 |
| Current | 110 (15.0) |  | 34.7 (12.1) | **0.001** |  | 42.4 (11.1) | **< 0.001** |
| ***P* for trend** |  |  |  | **0.008** |  |  | **0.01** |
| **BMI** |  |  |  |  |  |  |  |
| < 25 | 187 (31.6) |  | 37.8 (12.3) |  |  | 45.3 (11.2) |  |
| ≥ 25, < 30 | 213 (36.0) |  | 38.4 (11.6) | 0.48 |  | 46.3 (11.0) | 0.41 |
| ≥ 30 | 192 (32.4) |  | 38.2 (12.4) | 0.76 |  | 47.5 (11.8) | **0.03** |
| ***P* for trend** |  |  |  | 0.79 |  |  | **0.03** |
| **Etiology** |  |  |  |  |  |  |  |
| HBV + HCV | 186 (25.3) |  | 40.2 (12.2) |  |  | 48.2 (11.2) |  |
| Alcohol | 186 (25.3) |  | 37.8 (11.8) | 0.05 |  | 45.7 (11.8) | **0.02** |
| HBV + HCV + Alcohol | 175 (23.8) |  | 35.2 (11.9) | **< 0.001** |  | 43.2 (11.2) | **< 0.001** |
| NASH | 42 ( 5.7) |  | 40.2 (12.4) | 0.94 |  | 49.3 (10.5) | 0.59 |
| Other ^b^ | 146 (19.9) |  | 37.7 (11.9) | 0.08 |  | 47.6 (10.4) | 0.33 |
| **Child-Pugh score** |  |  |  |  |  |  |  |
| A | 584 (79.5) |  | 39.1 (12.0) |  |  | 46.8 (11.1) |  |
| B | 132 (18.0) |  | 33.2 (11.1) | **< 0.001** |  | 44.2 (11.7) | **0.02** |
| C | 18 ( 2.5) |  | 33.4 (12.1) | **0.03** |  | 44.4 (13.1) | 0.47 |
| ***P* for trend** |  |  |  | **< 0.001** |  |  | **0.03** |
| **Cirrhosis** |  |  |  |  |  |  |  |
| No | 287 (39.0) |  | 39.0 (12.3) |  |  | 47.8 (11.1) |  |
| Yes | 448 (61.0) |  | 37.2 (11.9) | 0.05 |  | 45.3 (11.4) | **0.003** |
| **Portal hypertension** |  |  |  |  |  |  |  |
| No | 494 (67.2) |  | 38.6 (12.0) |  |  | 46.7 (11.2) |  |
| Yes | 241 (32.8) |  | 36.4 (12.0) | **0.02** |  | 45.4 (11.6) | 0.17 |
| **Portal vein thrombosis** |  |  |  |  |  |  |  |
| No | 523 (71.2) |  | 39.5 (11.9) |  |  | 46.8 (11.1) |  |
| Yes | 212 (28.8) |  | 34.1 (11.7) | **< 0.001** |  | 44.9 (11.7) | **0.04** |
| **Histological grade** |  |  |  |  |  |  |  |
| Well | 129 (26.4) |  | 40.3 (11.9) |  |  | 48.4 (11.1) |  |
| Moderate | 222 (45.5) |  | 38.4 (12.3) | 0.14 |  | 46.9 (11.6) | 0.19 |
| Poor + undifferentiated | 137 (28.1) |  | 37.9 (12.3) | 0.13 |  | 45.7 (10.6) | **0.02** |
| ***P* for trend** |  |  |  | 0.12 |  |  | **0.02** |
| **NCCN tumor stage** |  |  |  |  |  |  |  |
| I | 102 (14.0) |  | 42.1 (10.7) |  |  | 50.5 (10.1) |  |
| II | 113 (15.5) |  | 41.6 (11.9) | 0.80 |  | 47.3 (11.6) | **0.046** |
| III | 293 (40.1) |  | 36.9 (12.0) | **< 0.001** |  | 45.1 (11.2) | **< 0.001** |
| IV | 223 (30.5) |  | 35.5 (12.0) | **< 0.001** |  | 45.5 (11.5) | **< 0.001** |
| ***P* for trend** |  |  |  | **< 0.001** |  |  | **< 0.001** |
| **Comorbidity** |  |  |  |  |  |  |  |
| No | 97 (13.2) |  | 38.1 (12.4) |  |  | 48.2 (10.4) |  |
| 1 | 171 (23.3) |  | 37.6 (12.7) | 0.69 |  | 45.7 (11.3) | 0.09 |
| 2 | 223 (30.3) |  | 40.4 (11.8) | 0.11 |  | 46.6 (10.8) | 0.27 |
| > 2 | 244 (33.2) |  | 35.7 (11.3) | 0.10 |  | 45.7 (12.1) | 0.14 |
| ***P* for trend** |  |  |  | 0.12 |  |  | 0.33 |
| **Prior treatment** |  |  |  |  |  |  |  |
| No | 543 (73.9) |  | 38.1 (12.2) |  |  | 46.1 (11.4) |  |
| Curative | 67 ( 9.1) |  | 40.4 (11.1) | 0.11 |  | 48.0 (10.6) | 0.23 |
| Palliative | 125 (17.0) |  | 35.8 (11.6) | 0.05 |  | 46.0 (11.2) | 0.91 |
| **Time since diagnosis ^c^** |  |  |  |  |  |  |  |
| < 1 month | 234 (31.8) |  | 39.2 (12.1) |  |  | 46.0 (11.5) |  |
| 1-3 months | 347 (47.2) |  | 37.5 (12.1) | 0.10 |  | 46.3 (11.5) | 0.68 |
| 3-6 months | 90 (12.2) |  | 36.8 (12.4) | 0.10 |  | 46.5 (11.1) | 0.71 |
| ≥ 6 months | 64 ( 8.7) |  | 36.8 (11.3) | 0.12 |  | 47.1 (10.4) | 0.56 |
| ***P* for trend** |  |  |  | 0.05 |  |  | 0.54 |
| **Years of diagnosis** |  |  |  |  |  |  |  |
| 1999-2002 | 106 (14.4) |  | 38.5 (11.8) |  |  | 47.4 (10.4) |  |
| 2003-2006 | 198 (26.9) |  | 37.7 (12.4) | 0.54 |  | 45.5 (11.5) | 0.21 |
| 2007-2009 | 226 (30.7) |  | 37.8 (12.5) | 0.58 |  | 46.7 (11.4) | 0.75 |
| 2010-2012 | 205 (27.9) |  | 37.9 (11.4) | 0.76 |  | 46.1 (11.5) | 0.43 |
| ***P* for trend** |  |  |  | 0.87 |  |  | 0.87 |
| **AFP (ng/ml)** |  |  |  |  |  |  |  |
| ≤ 6 | 108 (15.5) |  | 38.8 (11.8) |  |  | 48.7 (11.8) |  |
| > 6, ≤ 100 | 216 (30.9) |  | 40.1 (11.6) | 0.31 |  | 45.9 (11.2) | **0.02** |
| > 100, ≤ 1000 | 138 (19.8) |  | 37.6 (13.1) | 0.46 |  | 45.3 (12.0) | **0.02** |
| > 1000 | 236 (33.8) |  | 36.0 (11.6) | 0.06 |  | 46.0 (10.8) | **0.01** |
| ***P* for trend** |  |  |  | **0.002** |  |  | 0.06 |
| **CA19-9 (U/ml)** |  |  |  |  |  |  |  |
| ≤ 35 | 157 (45.2) |  | 39.3 (11.5) |  |  | 46.5 (11.6) |  |
| > 35, ≤ 100 | 103 (29.7) |  | 37.8 (11.8) | 0.32 |  | 45.2 (11.2) | 0.39 |
| > 100 | 87 (25.1) |  | 32.4 (11.8) | **< 0.001** |  | 44.0 (11.7) | 0.12 |
| ***P* for trend** |  |  |  | **< 0.001** |  |  | 0.11 |
| **ALT (U/L)** |  |  |  |  |  |  |  |
| ≤ 56 | 190 (52.1) |  | 38.4 (12.2) |  |  | 45.3 (11.7) |  |
| > 56, ≤ 100 | 94 (25.8) |  | 38.3 (11.7) | 0.91 |  | 47.0 (11.5) | 0.31 |
| > 100 | 81 (22.2) |  | 37.4 (12.4) | 0.59 |  | 46.6 (10.7) | 0.54 |
| ***P* for trend** |  |  |  | 0.67 |  |  | 0.44 |
| **AST (U/L)** |  |  |  |  |  |  |  |
| ≤ 46 | 68 (20.2) |  | 40.9 (11.5) |  |  | 45.3 (11.8) |  |
| > 46, ≤ 100 | 117 (34.8) |  | 39.2 (12.0) | 0.39 |  | 47.3 (11.9) | 0.25 |
| > 100 | 151 (44.9) |  | 36.2 (12.4) | **0.01** |  | 45.3 (11.1) | 0.99 |
| ***P* for trend** |  |  |  | **0.007** |  |  | 0.65 |
| **ALP (U/L)** |  |  |  |  |  |  |  |
| ≤ 126 | 119 (32.7) |  | 42.1 (11.2) |  |  | 48.2 (10.6) |  |
| > 126, ≤ 200 | 103 (28.3) |  | 38.9 (12.2) | 0.07 |  | 46.1 (11.4) | 0.21 |
| > 200 | 142 (39.0) |  | 34.3 (11.6) | **< 0.001** |  | 44.1 (11.7) | **0.008** |
| ***P* for trend** |  |  |  | **< 0.001** |  |  | **0.009** |
| **Total bilirubin (mg/dl)** |  |  |  |  |  |  |  |
| ≤ 1.0 | 235 (64.4) |  | 39.7 (12.2) |  |  | 47.2 (11.4) |  |
| > 1.0, ≤ 2.0 | 83 (22.7) |  | 35.9 (11.7) | **0.02** |  | 43.8 (11.6) | **0.02** |
| >2.0 | 47 (12.9) |  | 34.7 (11.6) | **0.01** |  | 44.7 (10.5) | 0.12 |
| ***P* for trend** |  |  |  | **0.002** |  |  | **0.03** |
| **Direct bilirubin (mg/dl)** |  |  |  |  |  |  |  |
| ≤ 0.4 | 207 (70.6) |  | 40.1 (11.6) |  |  | 47.5 (11.2) |  |
| > 0.4 | 86 (29.4) |  | 33.9 (11.4) | **< 0.001** |  | 42.9 (12.1) | **0.004** |
| **Serum albumin (g/dl)** |  |  |  |  |  |  |  |
| ≥ 3.5 | 262 (72.4) |  | 40.0 (12.2) |  |  | 47.3 (11.0) |  |
| ≥ 3.2, < 3.5 | 57 (15.7) |  | 36.0 (10.3) | **0.02** |  | 43.4 (11.5) | **0.02** |
| < 3.2 | 43 (11.9) |  | 30.4 (10.7) | **< 0.001** |  | 42.0 (12.2) | **0.01** |
| ***P* for trend** |  |  |  | **< 0.001** |  |  | **0.002** |
| **INR** |  |  |  |  |  |  |  |
| ≤ 1.2 | 198 (55.6) |  | 39.5 (12.3) |  |  | 46.9 (11.4) |  |
| > 1.2 | 158 (44.4) |  | 36.7 (11.8) | **0.03** |  | 45.1 (11.4) | 0.15 |
| **WBC (× 10^9^/L)** |  |  |  |  |  |  |  |
| 4-11 | 293 (79.8) |  | 38.8 (12.5) |  |  | 46.8 (11.2) |  |
| < 4 | 43 (11.7) |  | 38.9 (10.5) | 0.98 |  | 45.2 (10.8) | 0.31 |
| > 11 | 31 ( 8.4) |  | 31.6 (7.9) | **0.002** |  | 40.2 (13.1) | **0.008** |
| ***P* for trend** |  |  |  | **0.006** |  |  | **0.007** |
| **Lymphocytes (× 10^9^/L)** |  |  |  |  |  |  |  |
| < 1.0 | 99 (27.0) |  | 35.1 (10.6) |  |  | 44.2 (11.9) |  |
| ≥ 1.0 | 267 (73.0) |  | 39.4 (12.4) | **0.004** |  | 46.7 (11.2) | 0.07 |
| **Monocytes (× 10^9^/L)** |  |  |  |  |  |  |  |
| ≤ 0.7 | 257 (70.2) |  | 39.7 (12.2) |  |  | 46.5 (10.9) |  |
| > 0.7 | 109 (29.8) |  | 34.8 (11.3) | **< 0.001** |  | 45.0 (12.6) | 0.42 |
| **Neutrophils (× 10^9^/L)** |  |  |  |  |  |  |  |
| ≤ 7.3 | 316 (86.3) |  | 38.9 (12.2) |  |  | 46.7 (11.0) |  |
| > 7.3 | 50 (13.7) |  | 34.0 (10.5) | **0.004** |  | 41.8 (13.0) | **0.02** |
| **NLR** |  |  |  |  |  |  |  |
| ≤ 4.0 | 246 (67.2) |  | 39.9 (12.3) |  |  | 47.4 (10.6) |  |
| > 4.0 | 120 (32.8) |  | 35.0 (11.1) | **< 0.001** |  | 43.4 (12.5) | **0.007** |
| **LMR** |  |  |  |  |  |  |  |
| ≤ 2.9 | 233 (63.7) |  | 36.5 (11.7) |  |  | 44.9 (12.0) |  |
| > 2.9 | 133 (36.3) |  | 41.2 (12.2) | **< 0.001** |  | 48.1 (10.1) | **0.03** |

Abbreviations: AFP, alpha-fetoprotein; ALP, alkaline phosphatase ; ALT, alanine aminotransferase; AST, aspartate aminotransferase; BMI, body mass index; CA19-9, carbohydrate antigen 19-9; HBV, hepatitis B virus; HCV, hepatitis C virus; INR, international normalized ratio; LMR, lymphocyte-to-monocyte ratio; MCS, Mental Component Summary; NASH, non-alcoholic steatohepatitis; NCCN, National Comprehensive Cancer Network; NLR, neutrophil-to-Lymphocyte ratio; PCS, Physical Component Summary; SD, standard deviation; WBC, white blood cell.

^a^ Missing values not included: BMI (N = 143), Child-Pugh score (N = 1), Histological grade (N = 247), NCCN tumor stage (N = 4), AFP (N = 37), CA19-9 (N = 388), ALT (N = 370), AST (N = 399), alkaline phosphatase (N = 371), total bilirubin (N = 370), direct bilirubin (N = 442), serum albumin (N = 373), INR (N = 379), WBC (N = 368), lymphocytes (N = 369), monocytes (N = 369), neutrophils (N = 369). Percentages may not add up to 100% because of rounding.

^b^ Including cryptogenic (N = 128), poison (N = 6), autoimmune (N = 5), hemochromatosis (N = 5), primary biliary cirrhosis (N = 1), and estrogen (N = 1).

^c^ Interval between initial diagnosis and quality of life survey.
