# Supplementary material for: Elevated systemic inflammatory responses, factors associated with physical and mental quality of life, and prognosis of hepatocellular carcinoma
Source: Aging (Albany NY). 2020 Mar 7;12(5):4357–70. doi: 10.18632/aging.102889 (PMC7093167; doi:10.18632/aging.102889)
Supplement: Supplementary Table 2 [file aging-12-102889-s005..docx]

**Supplementary Table 2. Association between patient characteristics and low PCS/MCS scores (univariate and multivariate logistic regression analyses)**

| **Characteristic** | **PCS** | | | |  | **MCS** | | | |
| --- | --- | --- | --- | --- | --- | --- | --- | --- | --- |
|  | **Unadjusted**  **OR (95% CI)** | ***P* value** | **Adjusted**  **OR (95% CI)** | ***P* value** |  | **Unadjusted**  **OR (95% CI)** | ***P* value** | **Adjusted**  **OR (95% CI)** | ***P* value** |
| **Age, years** |  |  |  |  |  |  |  |  |  |
| < 55 | 1.00 (Ref) |  | 1.00 (Ref) |  |  | 1.00 (Ref) |  | 1.00 (Ref) |  |
| ≥ 55, < 65 | 0.97 (0.68-1.38) | 0.86 | 0.89 (0.59-1.35) | 0.59 |  | 0.89 (0.62-1.26) | 0.51 | 0.82 (0.55-1.24) | 0.35 |
| ≥ 65, < 75 | 0.64 (0.44-0.92) | **0.02** | 0.71 (0.46-1.09) | 0.12 |  | 0.78 (0.54-1.12) | 0.18 | 0.88 (0.57-1.35) | 0.55 |
| ≥ 75 | 0.69 (0.45-1.06) | 0.09 | 0.90 (0.54-1.52) | 0.70 |  | 0.51 (0.33-0.79) | **0.003** | 0.53 (0.31-0.90) | **0.02** |
| ***P*  for trend** |  | **0.01** |  | 0.38 |  |  | **0.004** |  | 0.05 |
| **Sex** |  |  |  |  |  |  |  |  |  |
| Male | 1.00 (Ref) |  | 1.00 (Ref) |  |  | 1.00 (Ref) |  | 1.00 (Ref) |  |
| Female | 1.21 (0.89-1.64) | 0.22 | 1.68 (1.15-2.44) | **0.007** |  | 1.44 (1.06-1.96) | **0.02** | 1.62 (1.11-2.36) | **0.01** |
| **Race/ethnicity** |  |  |  |  |  |  |  |  |  |
| Non-Hispanic white | 1.00 (Ref) |  | 1.00 (Ref) |  |  | 1.00 (Ref) |  | 1.00 (Ref) |  |
| Hispanic | 0.98 (0.68-1.41) | 0.92 | 1.08 (0.72-1.63) | 0.71 |  | 1.02 (0.71-1.46) | 0.93 | 1.27 (0.84-1.92) | 0.26 |
| African American | 1.93 (1.17-3.17) | **0.01** | 1.63 (0.94-2.84) | 0.08 |  | 1.59 (0.97-2.60) | 0.07 | 1.17 (0.68-2.01) | 0.58 |
| Asian | 1.02 (0.66-1.59) | 0.91 | 1.16 (0.69-1.96) | 0.57 |  | 1.12 (0.72-1.73) | 0.62 | 1.13 (0.67-1.90) | 0.66 |
| **Alcohol consumption** |  |  |  |  |  |  |  |  |  |
| Never | 1.00 (Ref) |  | 1.00 (Ref) |  |  | 1.00 (Ref) |  | 1.00 (Ref) |  |
| Former | 1.01 (0.76-1.35) | 0.94 | 0.95 (0.65-1.40) | 0.79 |  | 1.18 (0.89-1.58) | 0.25 | 1.04 (0.71-1.54) | 0.83 |
| Current | 0.60 (0.40-0.91) | **0.01** | 0.72 (0.44-1.17) | 0.19 |  | 0.74 (0.49-1.11) | 0.14 | 0.81 (0.49-1.32) | 0.40 |
| ***P*  for trend** |  | 0.05 |  | 0.21 |  |  | 0.44 |  | 0.46 |
| **Smoking status** |  |  |  |  |  |  |  |  |  |
| Never | 1.00 (Ref) |  | 1.00 (Ref) |  |  | 1.00 (Ref) |  | 1.00 (Ref) |  |
| Former | 1.04 (0.78-1.39) | 0.80 | 1.26 (0.89-1.79) | 0.19 |  | 0.95 (0.71-1.28) | 0.74 | 0.96 (0.68-1.36) | 0.82 |
| Current | 2.05 (1.35-3.13) | **< 0.001** | 2.18 (1.31-3.64) | **0.003** |  | 1.90 (1.26-2.88) | **0.002** | 1.47 (0.89-2.43) | 0.13 |
| ***P*  for trend** |  | **0.005** |  | **0.004** |  |  | **0.02** |  | 0.22 |
| **BMI** |  |  |  |  |  |  |  |  |  |
| < 25 | 1.00 (Ref) |  | 1.00 (Ref) |  |  | 1.00 (Ref) |  | 1.00 (Ref) |  |
| ≥ 25, < 30 | 0.91 (0.63-1.30) | 0.59 | 1.02 (0.67-1.53) | 0.93 |  | 0.87 (0.61-1.25) | 0.45 | 0.99 (0.66-1.48) | 0.97 |
| ≥ 30 | 0.95 (0.66-1.38) | 0.80 | 1.18 (0.76-1.84) | 0.46 |  | 0.71 (0.49-1.02) | 0.07 | 0.80 (0.52-1.23) | 0.31 |
| Unknown | 1.33 (0.89-1.98) | 0.17 | 1.86 (1.05-3.31) | **0.03** |  | 1.06 (0.71-1.58) | 0.78 | 1.27 (0.72-2.24) | 0.40 |
| ***P*  for trend** |  | 0.07 |  | **0.02** |  |  | 0.45 |  | 0.44 |
| **Etiology** |  |  |  |  |  |  |  |  |  |
| HBV + HCV | 1.00 (Ref) |  | 1.00 (Ref) |  |  | 1.00 (Ref) |  | 1.00 (Ref) |  |
| Alcohol | 0.68 (0.47-0.99) | **0.047** | 0.84 (0.52-1.35) | 0.47 |  | 0.64 (0.44-0.94) | **0.02** | 0.76 (0.47-1.22) | 0.26 |
| HBV + HCV + Alcohol | 1.36 (0.93-2.00) | 0.11 | 1.18 (0.74-1.90) | 0.48 |  | 1.61 (1.10-2.35) | **0.01** | 1.77 (1.10-2.85) | **0.02** |
| NASH | 0.73 (0.39-1.36) | 0.33 | 0.98 (0.49-1.97) | 0.95 |  | 0.55 (0.29-1.05) | 0.07 | 0.66 (0.33-1.33) | 0.24 |
| Other | 1.01 (0.68-1.50) | 0.97 | 1.18 (0.73-1.92) | 0.50 |  | 0.80 (0.54-1.19) | 0.27 | 0.88 (0.54-1.42) | 0.59 |
| **Child-Pugh score** |  |  |  |  |  |  |  |  |  |
| A | 1.00 (Ref) |  | 1.00 (Ref) |  |  | 1.00 (Ref) |  | 1.00 (Ref) |  |
| B | 2.19 (1.54-3.11) | **< 0.001** | 1.97 (1.33-2.92) | **< 0.001** |  | 1.47 (1.03-2.08) | **0.03** | 1.29 (0.88-1.89) | 0.20 |
| C | 2.71 (1.08-6.82) | **0.03** | 3.57 (1.31-9.73) | **0.01** |  | 1.41 (0.57-3.48) | 0.45 | 1.77 (0.67-4.69) | 0.25 |
| ***P*  for trend** |  | **< 0.001** |  | **< 0.001** |  |  | **0.04** |  | 0.10 |
| **Cirrhosis** |  |  |  |  |  |  |  |  |  |
| No | 1.00 (Ref) |  | 1.00 (Ref) |  |  | 1.00 (Ref) |  | 1.00 (Ref) |  |
| Yes | 1.26 (0.96-1.66) | 0.10 | 1.04 (0.73-1.48) | 0.84 |  | 1.40 (1.07-1.84) | **0.02** | 0.95 (0.67-1.36) | 0.79 |
| **Portal hypertension** |  |  |  |  |  |  |  |  |  |
| No | 1.00 (Ref) |  | 1.00 (Ref) |  |  | 1.00 (Ref) |  | 1.00 (Ref) |  |
| Yes | 1.28 (0.97-1.70) | 0.08 | 1.01 (0.69-1.48) | 0.97 |  | 1.22 (0.92-1.62) | 0.18 | 0.89 (0.61-1.30) | 0.55 |
| **Portal vein thrombosis** |  |  |  |  |  |  |  |  |  |
| No | 1.00 (Ref) |  | 1.00 (Ref) |  |  | 1.00 (Ref) |  | 1.00 (Ref) |  |
| Yes | 2.10 (1.56-2.83) | **< 0.001** | 1.52 (1.06-2.19) | **0.02** |  | 1.34 (0.99-1.80) | 0.06 | 0.90 (0.63-1.29) | 0.56 |
| **Histologic grade** |  |  |  |  |  |  |  |  |  |
| Well | 1.00 (Ref) |  | 1.00 (Ref) |  |  | 1.00 (Ref) |  | 1.00 (Ref) |  |
| Moderate | 1.18 (0.79-1.76) | 0.42 | 1.35 (0.87-2.08) | 0.18 |  | 1.36 (0.90-2.04) | 0.14 | 1.33 (0.86-2.06) | 0.20 |
| Poor + undifferentiated | 1.37 (0.88-2.13) | 0.17 | 1.12 (0.69-1.81) | 0.65 |  | 1.72 (1.10-2.67) | **0.02** | 1.55 (0.96-2.51) | 0.07 |
| ***P*  for trend** |  | **0.003** |  | 0.15 |  |  | **0.01** |  | 0.15 |
| **NCCN tumor stage** |  |  |  |  |  |  |  |  |  |
| I | 1.00 (Ref) |  | 1.00 (Ref) |  |  | 1.00 (Ref) |  | 1.00 (Ref) |  |
| II | 1.30 (0.79-2.13) | 0.30 | 1.07 (0.60-1.89) | 0.83 |  | 1.62 (0.99-2.66) | 0.05 | 1.25 (0.70-2.23) | 0.45 |
| III | 2.49 (1.64-3.79) | **< 0.001** | 1.78 (0.99-3.23) | 0.06 |  | 2.31 (1.52-3.51) | **< 0.001** | 1.82 (1.01-3.29) | **0.046** |
| IV | 2.85 (1.84-4.42) | **< 0.001** | 2.04 (1.12-3.72) | **0.02** |  | 2.10 (1.36-3.26) | **< 0.001** | 1.60 (0.88-2.92) | 0.12 |
| ***P*  for trend** |  | **< 0.001** |  | **0.03** |  |  | **< 0.001** |  | 0.13 |
| **Comorbidity** |  |  |  |  |  |  |  |  |  |
| No | 1.00 (Ref) |  | 1.00 (Ref) |  |  | 1.00 (Ref) |  | 1.00 (Ref) |  |
| 1 | 0.94 (0.60-1.48) | 0.78 | 1.03 (0.62-1.69) | 0.91 |  | 1.52 (0.96-2.38) | 0.07 | 1.78 (1.07-2.93) | **0.03** |
| 2 | 0.60 (0.39-0.92) | **0.02** | 0.80 (0.49-1.30) | 0.37 |  | 1.21 (0.79-1.86) | 0.38 | 1.77 (1.08-2.88) | **0.02** |
| > 2 | 1.30 (0.85-2.00) | 0.23 | 2.12 (1.28-3.51) | **0.004** |  | 1.31 (0.86-2.01) | 0.21 | 1.98 (1.20-3.27) | **0.008** |
| ***P*  for trend** |  | 0.24 |  | **0.001** |  |  | 0.60 |  | **0.03** |
| **Prior treatment** |  |  |  |  |  |  |  |  |  |
| No | 1.00 (Ref) |  | 1.00 (Ref) |  |  | 1.00 (Ref) |  | 1.00 (Ref) |  |
| Curative | 0.82 (0.52-1.30) | 0.41 | 1.65 (0.96-2.85) | 0.07 |  | 0.79 (0.50-1.24) | 0.30 | 0.90 (0.53-1.55) | 0.72 |
| Palliative | 1.66 (1.16-2.39) | **0.006** | 1.71 (1.14-2.56) | **0.009** |  | 1.02 (0.71-1.46) | 0.93 | 0.79 (0.53-1.17) | 0.25 |
| **Time since diagnosis ^a^** |  |  |  |  |  |  |  |  |  |
| < 1 month | 1.00 (Ref) |  | 1.00 (Ref) |  |  | 1.00 (Ref) |  | 1.00 (Ref) |  |
| 1-3 months | 1.31 (0.96-1.77) | 0.09 | 1.28 (0.91-1.80) | 0.15 |  | 0.93 (0.68-1.26) | 0.62 | 0.88 (0.63-1.23) | 0.46 |
| 3-6 months | 1.66 (1.06-2.60) | **0.03** | 1.35 (0.77-2.34) | 0.29 |  | 0.87 (0.55-1.37) | 0.56 | 0.74 (0.43-1.29) | 0.29 |
| ≥ 6 months | 1.61 (0.97-2.68) | 0.07 | 1.51 (0.80-2.87) | 0.20 |  | 0.94 (0.57-1.55) | 0.82 | 0.86 (0.46-1.60) | 0.63 |
| ***P*  for trend** |  | **0.01** |  | 0.14 |  |  | 0.65 |  | 0.42 |
| **Years of diagnosis** |  |  |  |  |  |  |  |  |  |
| 1999-2002 | 1.00 (Ref) |  | 1.00 (Ref) |  |  | 1.00 (Ref) |  | 1.00 (Ref) |  |
| 2003-2006 | 1.06 (0.69-1.64) | 0.79 | 1.01 (0.63-1.62) | 0.97 |  | 1.18 (0.77-1.81) | 0.45 | 1.27 (0.80-2.02) | 0.32 |
| 2007-2009 | 0.99 (0.65-1.51) | 0.96 | 1.00 (0.63-1.61) | 0.99 |  | 0.94 (0.62-1.43) | 0.78 | 0.93 (0.59-1.48) | 0.76 |
| 2010-2012 | 0.89 (0.58-1.37) | 0.60 | 0.81 (0.50-1.32) | 0.40 |  | 1.05 (0.69-1.61) | 0.81 | 1.16 (0.71-1.87) | 0.56 |
| ***P* for trend** |  | 0.43 |  | 0.33 |  |  | 0.79 |  | 0.98 |
| **AFP (ng/ml)** |  |  |  |  |  |  |  |  |  |
| ≤ 6 | 1.00 (Ref) |  | 1.00 (Ref) |  |  | 1.00 (Ref) |  | 1.00 (Ref) |  |
| > 6, ≤ 100 | 0.89 (0.58-1.36) | 0.59 | 0.58 (0.36-0.93) | **0.02** |  | 1.49 (0.96-2.29) | 0.07 | 1.14 (0.71-1.83) | 0.59 |
| > 100, ≤ 1000 | 1.40 (0.88-2.24) | 0.16 | 0.94 (0.56-1.58) | 0.81 |  | 1.58 (0.99-2.54) | 0.06 | 1.07 (0.63-1.80) | 0.80 |
| > 1000 | 1.72 (1.13-2.62) | **0.01** | 1.04 (0.64-1.69) | 0.87 |  | 1.74 (1.13-2.67) | **0.01** | 1.10 (0.68-1.78) | 0.71 |
| ***P*  for trend** |  | **< 0.001** |  | 0.10 |  |  | **0.02** |  | 0.89 |
| **CA19-9 (U/ml)** |  |  |  |  |  |  |  |  |  |
| ≤ 35 | 1.00 (Ref) |  | 1.00 (Ref) |  |  | 1.00 (Ref) |  | 1.00 (Ref) |  |
| > 35, ≤ 100 | 1.21 (0.77-1.91) | 0.40 | 1.03 (0.60-1.77) | 0.92 |  | 1.20 (0.76-1.90) | 0.43 | 1.09 (0.64-1.86) | 0.74 |
| > 100 | 2.78 (1.68-4.61) | **< 0.001** | 1.97 (1.08-3.62) | **0.03** |  | 1.42 (0.87-2.30) | 0.16 | 1.17 (0.65-2.11) | 0.59 |
| ***P*  for trend** |  | **< 0.001** |  | **0.04** |  |  | 0.15 |  | 0.58 |
| **ALT (U/L)** |  |  |  |  |  |  |  |  |  |
| ≤ 56 | 1.00 (Ref) |  | 1.00 (Ref) |  |  | 1.00 (Ref) |  | 1.00 (Ref) |  |
| > 56, ≤ 100 | 1.08 (0.69-1.70) | 0.74 | 1.12 (0.65-1.92) | 0.69 |  | 0.86 (0.54-1.35) | 0.51 | 0.65 (0.38-1.12) | 0.12 |
| > 100 | 1.07 (0.67-1.72) | 0.78 | 0.98 (0.55-1.76) | 0.95 |  | 0.93 (0.57-1.49) | 0.75 | 0.70 (0.39-1.25) | 0.22 |
| ***P*  for trend** |  | 0.74 |  | 0.99 |  |  | 0.66 |  | 0.16 |
| **AST (U/L)** |  |  |  |  |  |  |  |  |  |
| ≤ 46 | 1.00 (Ref) |  | 1.00 (Ref) |  |  | 1.00 (Ref) |  | 1.00 (Ref) |  |
| > 46, ≤ 100 | 1.19 (0.68-2.07) | 0.54 | 0.76 (0.39-1.48) | 0.42 |  | 0.78 (0.45-1.35) | 0.37 | 0.58 (0.30-1.12) | 0.11 |
| > 100 | 1.85 (1.09-3.14) | **0.02** | 0.92 (0.45-1.88) | 0.81 |  | 1.08 (0.64-1.82) | 0.77 | 0.51 (0.25-1.06) | 0.07 |
| ***P*  for trend** |  | **0.01** |  | 0.92 |  |  | 0.54 |  | 0.09 |
| **ALP (U/L)** |  |  |  |  |  |  |  |  |  |
| ≤ 126 | 1.00 (Ref) |  | 1.00 (Ref) |  |  | 1.00 (Ref) |  | 1.00 (Ref) |  |
| > 126, ≤ 200 | 1.61 (0.98-2.62) | 0.06 | 1.47 (0.84-2.60) | 0.18 |  | 1.39 (0.86-2.26) | 0.18 | 1.31 (0.74-2.34) | 0.35 |
| > 200 | 3.04 (1.92-4.84) | **< 0.001** | 2.70 (1.49-4.89) | **0.001** |  | 1.71 (1.08-2.69) | **0.02** | 1.41 (0.77-2.59) | 0.27 |
| ***P*  for trend** |  | **< 0.001** |  | **0.001** |  |  | **0.02** |  | 0.27 |
| **Total bilirubin (mg/dl)** |  |  |  |  |  |  |  |  |  |
| ≤ 1.0 | 1.00 (Ref) |  | 1.00 (Ref) |  |  | 1.00 (Ref) |  | 1.00 (Ref) |  |
| > 1.0, ≤ 2.0 | 1.57 (0.99-2.48) | 0.05 | 1.42 (0.81-2.47) | 0.22 |  | 1.74 (1.09-2.79) | **0.02** | 1.71 (0.97-3.00) | 0.06 |
| >2.0 | 2.05 (1.15-3.65) | **0.01** | 1.08 (0.46-2.51) | 0.87 |  | 1.51 (0.85-2.66) | 0.16 | 1.20 (0.51-2.79) | 0.68 |
| ***P*  for trend** |  | **0.005** |  | 0.50 |  |  | **0.04** |  | 0.26 |
| **Direct bilirubin (mg/dl)** |  |  |  |  |  |  |  |  |  |
| ≤ 0.4 | 1.00 (Ref) |  | 1.00 (Ref) |  |  | 1.00 (Ref) |  | 1.00 (Ref) |  |
| > 0.4 | 2.30 (1.44-3.68) | **< 0.001** | 1.67 (0.86-3.24) | 0.13 |  | 1.94 (1.20-3.12) | **0.007** | 2.23 (1.15-4.32) | **0.02** |
| **Serum albumin (g/dl)** |  |  |  |  |  |  |  |  |  |
| ≥ 3.5 | 1.00 (Ref) |  | 1.00 (Ref) |  |  | 1.00 (Ref) |  | 1.00 (Ref) |  |
| ≥ 3.2, < 3.5 | 1.74 (1.04-2.92) | **0.04** | 2.11 (1.12-3.98) | **0.02** |  | 1.62 (0.95-2.78) | 0.08 | 1.77 (0.92-3.40) | 0.09 |
| < 3.2 | 4.60 (2.44-8.65) | **< 0.001** | 5.83 (2.38-14.28) | **< 0.001** |  | 2.28 (1.23-4.20) | **0.009** | 2.75 (1.16-6.48) | **0.02** |
| ***P*  for trend** |  | **< 0.001** |  | **< 0.001** |  |  | **0.003** |  | **0.01** |
| **INR** |  |  |  |  |  |  |  |  |  |
| ≤ 1.2 | 1.00 (Ref) |  | 1.00 (Ref) |  |  | 1.00 (Ref) |  | 1.00 (Ref) |  |
| > 1.2 | 1.51 (1.03-2.22) | **0.04** | 1.24 (0.76-2.01) | 0.39 |  | 1.17 (0.80-1.72) | 0.42 | 0.91 (0.55-1.49) | 0.71 |
| **WBC (× 10^9^/L)** |  |  |  |  |  |  |  |  |  |
| 4-11 | 1.00 (Ref) |  | 1.00 (Ref) |  |  | 1.00 (Ref) |  | 1.00 (Ref) |  |
| < 4 | 0.92 (0.52-1.65) | 0.79 | 1.08 (0.55-2.13) | 0.83 |  | 1.29 (0.73-2.30) | 0.38 | 1.30 (0.66-2.56) | 0.45 |
| > 11 | 2.78 (1.40-5.50) | **0.003** | 3.19 (1.46-6.97) | **0.004** |  | 2.56 (1.23-5.34) | **0.01** | 2.72 (1.20-6.15) | **0.02** |
| ***P*  for trend** |  | **0.02** |  | **0.009** |  |  | **0.01** |  | **0.02** |
| **Lymphocytes (× 10^9^/L)** |  |  |  |  |  |  |  |  |  |
| ≥ 1.0 | 1.00 (Ref) |  | 1.00 (Ref) |  |  | 1.00 (Ref) |  | 1.00 (Ref) |  |
| < 1.0 | 1.59 (1.05-2.42) | **0.03** | 1.83 (1.11-3.02) | **0.02** |  | 1.49 (0.97-2.29) | 0.07 | 1.64 (0.99-2.72) | 0.05 |
| **Monocytes (× 10^9^/L)** |  |  |  |  |  |  |  |  |  |
| ≤ 0.7 | 1.00 (Ref) |  | 1.00 (Ref) |  |  | 1.00 (Ref) |  | 1.00 (Ref) |  |
| > 0.7 | 2.08 (1.37-3.16) | **< 0.001** | 2.19 (1.35-3.54) | **0.001** |  | 1.30 (0.86-1.98) | 0.22 | 1.27 (0.79-2.04) | 0.33 |
| **Neutrophils (× 10^9^/L)** |  |  |  |  |  |  |  |  |  |
| ≤ 7.3 | 1.00 (Ref) |  | 1.00 (Ref) |  |  | 1.00 (Ref) |  | 1.00 (Ref) |  |
| > 7.3 | 2.44 (1.40-4.25) | **0.002** | 2.67 (1.38-5.17) | **0.004** |  | 2.03 (1.15-3.60) | **0.02** | 2.57 (1.31-5.07) | **0.006** |
| **NLR** |  |  |  |  |  |  |  |  |  |
| ≤ 4.0 | 1.00 (Ref) |  | 1.00 (Ref) |  |  | 1.00 (Ref) |  | 1.00 (Ref) |  |
| > 4.0 | 2.14 (1.43-3.20) | **< 0.001** | 2.14 (1.30-3.53) | **0.003** |  | 1.65 (1.09-2.48) | **0.02** | 1.88 (1.14-3.12) | **0.01** |
| **LMR** |  |  |  |  |  |  |  |  |  |
| ≤ 2.9 | 1.00 (Ref) |  | 1.00 (Ref) |  |  | 1.00 (Ref) |  | 1.00 (Ref) |  |
| > 2.9 | 0.51 (0.34-0.76) | **< 0.001** | 0.53 (0.33-0.86) | **0.009** |  | 0.61 (0.41-0.90) | **0.01** | 0.63 (0.39-1.02) | **0.06** |

Abbreviations: AFP, alpha-fetoprotein; ALP, alkaline phosphatase ; ALT, alanine aminotransferase; AST, aspartate aminotransferase; BMI, body mass index; CA19-9, carbohydrate antigen 19-9; HBV, hepatitis B virus; HCV, hepatitis C virus; INR, international normalized ratio; LMR, lymphocyte-to-monocyte ratio; MCS, Mental Component Summary; NASH, non-alcoholic steatohepatitis; NCCN, National Comprehensive Cancer Network; NLR, neutrophil-to-Lymphocyte ratio; PCS, Physical Component Summary; OR, odds ratio; WBC, white blood cell.

^a^ Interval between initial diagnosis and quality of life survey.
