# Supplementary material for: Elevated systemic inflammatory responses, factors associated with physical and mental quality of life, and prognosis of hepatocellular carcinoma
Source: Aging (Albany NY). 2020 Mar 7;12(5):4357–70. doi: 10.18632/aging.102889 (PMC7093167; doi:10.18632/aging.102889)
Supplement: Supplementary Table 3 [file aging-12-102889-s002..docx]

**Supplementary table 3. Association between patient characteristics and low PCS score, stratified by race**

| **Characteristic** | **Non-Hispanic white (N = 469)** | |  | **Hispanic (N = 128)** | |  | **African American (N = 62)** | |  | **Asian (N =76)** | |
| --- | --- | --- | --- | --- | --- | --- | --- | --- | --- | --- | --- |
|  | **Unadjusted** | **Adjusted ^a^** |  | **Unadjusted** | **Adjusted ^a^** |  | **Unadjusted** | **Adjusted ^a^** |  | **Unadjusted** | **Adjusted ^a^** |
|  | **OR(95% CI)** | **OR(95% CI)** |  | **OR(95% CI)** | **OR(95% CI)** |  | **OR(95% CI)** | **OR(95% CI)** |  | **OR(95% CI)** | **OR(95% CI)** |
| **Age, years** |  |  |  |  |  |  |  |  |  |  |  |
| < 55 | 1.00(Ref) | 1.00(Ref) |  | 1.00(Ref) | 1.00(Ref) |  | 1.00(Ref) | 1.00(Ref) |  | 1.00(Ref) | 1.00(Ref) |
| ≥ 55, < 65 | 1.06(0.67-1.67) | 0.93(0.55-1.55) |  | 0.81(0.31-2.10) | 0.59(0.17-2.08) |  | 1.40(0.49-4.04) | 0.63(0.08-4.88) |  | 0.59(0.21-1.65) | 0.67(0.14-3.08) |
| ≥ 65, < 75 | 0.57(0.36-0.91) | 0.63(0.37-1.08) |  | 0.68(0.25-1.81) | 0.68(0.18-2.62) |  | 4.97(1.12-22.10) | 4.81(0.24-97.81) |  | 0.43(0.14-1.33) | 2.12(0.30-15.11) |
| ≥ 75 | 0.76(0.45-1.28) | 1.03(0.54-1.97) |  | 0.62(0.21-1.81) | 0.52(0.12-2.20) |  | 2.28(0.17-30.56) | NA ^b^ |  | 0.38(0.09-1.57) | 0.32(0.01-7.19) |
| **Sex** |  |  |  |  |  |  |  |  |  |  |  |
| Male | 1.00(Ref) | 1.00(Ref) |  | 1.00(Ref) | 1.00(Ref) |  | 1.00(Ref) | 1.00(Ref) |  | 1.00(Ref) | 1.00(Ref) |
| Female | 0.90(0.61-1.32) | 1.13(0.71-1.79) |  | 3.59(1.64-7.87) | 4.43(1.46-13.47) |  | 1.93(0.62-6.01) | NA |  | 1.05(0.43-2.57) | 2.72(0.51-14.40) |
| **Smoking status** |  |  |  |  |  |  |  |  |  |  |  |
| Never | 1.00(Ref) | 1.00(Ref) |  | 1.00(Ref) | 1.00(Ref) |  | 1.00(Ref) | 1.00(Ref) |  | 1.00(Ref) | 1.00(Ref) |
| Former | 0.95(0.65-1.39) | 1.16(0.75-1.79) |  | 0.87(0.45-1.67) | 1.12(0.47-2.65) |  | 1.94(0.64-5.85) | 32.69(0.98-1095.23) |  | 2.16(0.86-5.39) | 19.93(3.49-113.98) |
| Current | 2.54(1.51-4.29) | 2.71(1.47-4.99) |  | 0.46(0.10-2.22) | 1.84(0.27-12.36) |  | 3.33(0.90-12.35) | NA |  | 0.60(0.16-2.26) | 0.35(0.03-3.64) |
| **Etiology** |  |  |  |  |  |  |  |  |  |  |  |
| Alcohol | 1.00(Ref) | 1.00(Ref) |  | 1.00(Ref) | 1.00(Ref) |  | 1.00(Ref) | 1.00(Ref) |  | 1.00(Ref) | 1.00(Ref) |
| HBV + HCV | 1.40(0.85-2.30) | 1.13(0.64-2.00) |  | 2.17(0.86-5.44) | 2.02(0.63-6.47) |  | 1.25(0.21-7.57) | 4.09(0.07-234.24) |  | 0.80(0.04-16.88) | 0.01(0.00-3.30) |
| HBV + HCV + Alcohol | 1.95(1.22-3.11) | 1.38(0.81-2.36) |  | 1.35(0.51-3.54) | 1.01(0.33-3.08) |  | 1.51(0.27-8.42) | NA |  | 1.39(0.06-32.59) | NA |
| NASH | 1.14(0.54-2.38) | 1.02(0.47-2.25) |  | 1.04(0.32-3.36) | 1.63(0.37-7.17) |  | NA | NA |  | NA | NA |
| Other ^c^ | 1.40(0.88-2.24) | 1.43(0.85-2.43) |  | 1.93(0.79-4.71) | 1.56(0.49-4.94) |  | 0.50(0.07-3.76) | 0.19(0.00-16.56) |  | 1.92(0.07-52.12) | 1.13(0.00-295.23) |
| **Child-Pugh score** |  |  |  |  |  |  |  |  |  |  |  |
| A | 1.00(Ref) | 1.00(Ref) |  | 1.00(Ref) | 1.00(Ref) |  | 1.00(Ref) | 1.00(Ref) |  | 1.00(Ref) | 1.00(Ref) |
| B | 2.14(1.37-3.36) | 1.99(1.21-3.28) |  | 2.10(0.95-4.61) | 3.50(1.35-9.05) |  | 1.21(0.40-3.67) | 2.74(0.18-41.87) |  | 4.77(1.40-16.28) | 16.68(2.45-113.78) |
| C | 2.79(1.03-7.55) | 3.73(1.25-11.08) |  | NA | NA |  | NA | NA |  | NA | NA |
| **Portal vein thrombosis** |  |  |  |  |  |  |  |  |  |  |  |
| No | 1.00(Ref) | 1.00(Ref) |  | 1.00(Ref) | 1.00(Ref) |  | 1.00(Ref) | 1.00(Ref) |  | 1.00(Ref) | 1.00(Ref) |
| Yes | 1.97(1.36-2.85) | 1.49(0.96-2.33) |  | 2.30(1.03-5.12) | 1.22(0.41-3.63) |  | 1.60(0.58-4.42) | 10.55(0.63-175.86) |  | 3.50(1.36-8.99) | 5.81(1.00-33.86) |
| **NCCN tumor stage** |  |  |  |  |  |  |  |  |  |  |  |
| I | 1.00(Ref) | 1.00(Ref) |  | 1.00(Ref) | 1.00(Ref) |  | 1.00(Ref) | 1.00(Ref) |  | 1.00(Ref) | 1.00(Ref) |
| II | 1.27(0.68-2.39) | 1.15(0.59-2.25) |  | 1.36(0.46-4.05) | 1.13(0.30-4.26) |  | 1.00(0.16-6.37) | NA |  | 1.38(0.24-7.98) | 1.84(0.15-22.42) |
| III | 2.28(1.33-3.92) | 1.78(0.98-3.25) |  | 2.51(1.02-6.19) | 2.15(0.66-7.02) |  | 2.49(0.52-11.90) | NA |  | 4.73(1.16-19.35) | 10.75(0.97-119.29) |
| IV | 2.36(1.34-4.18) | 1.79(0.93-3.41) |  | 2.96(1.15-7.63) | 3.22(0.96-10.81) |  | 2.10(0.46-9.52) | NA |  | 9.03(2.12-38.43) | 55.25(4.86-628.46) |
| **Comorbidity** |  |  |  |  |  |  |  |  |  |  |  |
| No | 1.00(Ref) | 1.00(Ref) |  | 1.00(Ref) | 1.00(Ref) |  | 1.00(Ref) | 1.00(Ref) |  | 1.00(Ref) | 1.00(Ref) |
| 1 | 1.65(0.89-3.04) | 1.72(0.89-3.34) |  | 0.30(0.09-1.02) | 0.22(0.05-0.98) |  | 1.16(0.30-4.45) | 0.10(0.01-1.25) |  | 0.29(0.09-0.96) | 0.29(0.04-2.13) |
| 2 | 0.84(0.47-1.52) | 0.99(0.52-1.88) |  | 0.17(0.05-0.53) | 0.15(0.03-0.69) |  | 1.45(0.40-5.31) | 0.63(0.04-10.93) |  | 0.49(0.16-1.49) | 0.85(0.11-6.65) |
| > 2 | 1.76(0.98-3.14) | 2.35(1.23-4.48) |  | 0.55(0.19-1.62) | 0.66(0.17-2.54) |  | 13.06(2.14-79.73) | 4.92(0.23-104.11) |  | 0.54(0.14-2.10) | 1.11(0.07-18.98) |
| **Prior treatment** |  |  |  |  |  |  |  |  |  |  |  |
| No | 1.00(Ref) | 1.00(Ref) |  | 1.00(Ref) | 1.00(Ref) |  | 1.00(Ref) | 1.00(Ref) |  | 1.00(Ref) | 1.00(Ref) |
| Curative | 1.02(0.59-1.78) | 2.20(1.13-4.28) |  | 0.17(0.02-1.57) | 0.31(0.02-4.22) |  | 0.47(0.13-1.66) | 41.92(1.31-1345.98) |  | 0.48(0.10-2.35) | 10.46(0.80-137.53) |
| Palliative | 1.79(1.15-2.77) | 1.62(1.00-2.64) |  | 1.39(0.54-3.60) | 0.86(0.28-2.69) |  | 0.85(0.25-2.91) | 2.27(0.21-25.19) |  | 2.63(0.65-10.57) | 6.39(0.68-60.34) |
| **CA 19-9 (U/ml)** |  |  |  |  |  |  |  |  |  |  |  |
| ≤ 35 | 1.00(Ref) | 1.00(Ref) |  | 1.00(Ref) | 1.00(Ref) |  | 1.00(Ref) | 1.00(Ref) |  | 1.00(Ref) | 1.00(Ref) |
| > 35, ≤ 100 | 0.93(0.53-1.65) | 0.63(0.32-1.23) |  | 4.69(1.53-14.39) | 1.77(0.23-13.53) |  | 2.95(0.43-20.33) | NA |  | 0.30(0.05-1.60) | NA |
| > 100 | 2.81(1.49-5.28) | 1.76(0.82-3.78) |  | 3.21(0.86-11.92) | 56.49(2.92-1092.43) |  | 4.14(0.36-47.96) | NA |  | 2.30(0.56-9.52) | NA |
| **ALP (U/L)** |  |  |  |  |  |  |  |  |  |  |  |
| ≤ 126 | 1.00(Ref) | 1.00(Ref) |  | 1.00(Ref) | 1.00(Ref) |  | 1.00(Ref) | 1.00(Ref) |  | 1.00(Ref) | 1.00(Ref) |
| > 126, ≤ 200 | 1.40(0.77-2.53) | 1.06(0.54-2.08) |  | 2.73(0.87-8.57) | 3.71(0.60-23.17) |  | 2.80(0.32-24.17) | NA |  | 1.38(0.17-11.00) | NA |
| > 200 | 3.22(1.81-5.73) | 2.91(1.39-6.07) |  | 2.28(0.79-6.61) | 14.35(1.55-133.32) |  | 1.95(0.43-8.82) | NA |  | 8.39(1.03-68.36) | NA |
| **Direct bilirubin (mg/dl)** |  |  |  |  |  |  |  |  |  |  |  |
| ≤ 0.4 | 1.00(Ref) | 1.00(Ref) |  | 1.00(Ref) | 1.00(Ref) |  | 1.00(Ref) | 1.00(Ref) |  | 1.00(Ref) | 1.00(Ref) |
| > 0.4 | 2.72(1.48-4.99) | 2.45(1.07-5.56) |  | 1.53(0.52-4.48) | 8.65(0.49-151.89) |  | 0.73(0.17-3.11) | NA |  | 6.75(0.93-49.23) | NA |
| **Serum albumin (g/dl)** |  |  |  |  |  |  |  |  |  |  |  |
| ≥ 3.5 | 1.00(Ref) | 1.00(Ref) |  | 1.00(Ref) | 1.00(Ref) |  | 1.00(Ref) | 1.00(Ref) |  | 1.00(Ref) | 1.00(Ref) |
| ≥ 3.2, < 3.5 | 2.02(1.07-3.79) | 2.51(1.17-5.39) |  | 0.70(0.20-2.45) | 1.42(0.19-10.43) |  | 2.04(0.45-9.38) | NA |  | 1.72(0.07-45.27) | NA |
| < 3.2 | 5.76(2.60-12.76) | 6.51(2.09-20.22) |  | 2.79(0.77-10.08) | 23.51(1.61-342.53) |  | 4.20(0.36-49.25) | NA |  | 1.72(0.07-45.27) | NA |
| **WBC (× 10^9^/L)** |  |  |  |  |  |  |  |  |  |  |  |
| 4-11 | 1.00(Ref) | 1.00(Ref) |  | 1.00(Ref) | 1.00(Ref) |  | 1.00(Ref) | 1.00(Ref) |  | 1.00(Ref) | 1.00(Ref) |
| < 4 | 1.11(0.57-2.14) | 1.21(0.55-2.67) |  | 0.37(0.08-1.74) | 0.46(0.02-9.97) |  | 0.25(0.02-3.41) | NA |  | NA | NA |
| > 11 | 2.21(0.94-5.20) | 2.64(1.00-6.95) |  | 4.01(0.89-18.12) | 20.64(1.49-286.54) |  | 2.56(0.40-16.30) | NA |  | NA | NA |
| **Lymphocyte (× 10^9^/L)** |  |  |  |  |  |  |  |  |  |  |  |
| ≥ 1.0 | 1.00(Ref) | 1.00(Ref) |  | 1.00(Ref) | 1.00(Ref) |  | 1.00(Ref) | 1.00(Ref) |  | 1.00(Ref) | 1.00(Ref) |
| < 1.0 | 1.35(0.82-2.23) | 1.44(0.79-2.60) |  | 1.50(0.55-4.08) | 1.27(0.29-5.55) |  | 2.80(0.57-13.75) | NA |  | 22.12(1.86-262.34) | NA |
| **Monocyte (× 10^9^/L)** |  |  |  |  |  |  |  |  |  |  |  |
| ≤ 0.7 | 1.00(Ref) | 1.00(Ref) |  | 1.00(Ref) | 1.00(Ref) |  | 1.00(Ref) | 1.00(Ref) |  | 1.00(Ref) | 1.00(Ref) |
| > 0.7 | 1.91(1.16-3.15) | 2.21(1.26-3.88) |  | 2.24(0.79-6.40) | 2.75(0.51-14.84) |  | 3.17(0.79-12.73) | NA |  | 1.72(0.16-18.81) | NA |
| **Neutrophil (× 10^9^/L)** |  |  |  |  |  |  |  |  |  |  |  |
| ≤ 7.3 | 1.00(Ref) | 1.00(Ref) |  | 1.00(Ref) | 1.00(Ref) |  | 1.00(Ref) | 1.00(Ref) |  | 1.00(Ref) | 1.00(Ref) |
| > 7.3 | 2.02(1.04-3.93) | 2.39(1.07-5.33) |  | 4.42(0.99-19.77) | 22.35(1.67-299.38) |  | 2.80(0.57-13.75) | NA |  | 1.66(0.06-43.15) | NA |
| **NLR** |  |  |  |  |  |  |  |  |  |  |  |
| ≤ 4.0 | 1.00(Ref) | 1.00(Ref) |  | 1.00(Ref) | 1.00(Ref) |  | 1.00(Ref) | 1.00(Ref) |  | 1.00(Ref) | 1.00(Ref) |
| > 4.0 | 1.75(1.08-2.84) | 1.72(0.94-3.13) |  | 2.53(0.92-6.96) | 2.13(0.42-10.92) |  | 2.77(0.72-10.65) | NA |  | 22.12(1.86-262.34) | NA |
| **LMR** |  |  |  |  |  |  |  |  |  |  |  |
| ≤ 2.9 | 1.00(Ref) | 1.00(Ref) |  | 1.00(Ref) | 1.00(Ref) |  | 1.00(Ref) | 1.00(Ref) |  | 1.00(Ref) | 1.00(Ref) |
| > 2.9 | 0.60(0.37-0.99) | 0.60(0.33-1.09) |  | 0.61(0.25-1.48) | 2.91(0.67-12.56) |  | 0.12(0.03-0.56) | NA |  | 0.24(0.04-1.31) | NA |

Abbreviations: ALP, alkaline phosphatase; CA19-9, carbohydrate antigen 19-9; CI, confidence Interval; HBV, hepatitis B virus; HCV, hepatitis C virus; LMR, lymphocyte-to-monocyte ratio; NASH, non-alcoholic steatohepatitis; NCCN, National Comprehensive Cancer Network; NLR, neutrophil-to-lymphocyte ratio; OR, odds ratio; PCS, Physical Component Summary; WBC, white blood cell.

^a^ Adjusted for sex, age, smoking, etiology, Child-Pugh score, portal vein thrombosis, cancer stage, comorbidity, and prior treatment if appropriate.

^b^ Data were not available because of small sample size.

^c^ Including cryptogenic (N = 128), poison (N = 6), autoimmune (N = 5), hemochromatosis (N = 5), primary biliary cirrhosis (N = 1), and estrogen (N = 1).
