# Supplementary material for: Elevated systemic inflammatory responses, factors associated with physical and mental quality of life, and prognosis of hepatocellular carcinoma
Source: Aging (Albany NY). 2020 Mar 7;12(5):4357–70. doi: 10.18632/aging.102889 (PMC7093167; doi:10.18632/aging.102889)
Supplement: Supplementary Table 4 [file aging-12-102889-s004..docx]

**Supplementary table 4. Association between patient characteristics and low MCS score, stratified by race**

| **Characteristic** | **Non-Hispanic white (N = 469)** | |  | **Hispanic (N = 128)** | |  | **African American (N = 62)** | |  | **Asian (N =76)** | |
| --- | --- | --- | --- | --- | --- | --- | --- | --- | --- | --- | --- |
|  | **Unadjusted** | **Adjusted ^a^** |  | **Unadjusted** | **Adjusted ^a^** |  | **Unadjusted** | **Adjusted ^a^** |  | **Unadjusted** | **Adjusted ^a^** |
|  | **OR(95% CI)** | **OR(95% CI)** |  | **OR(95% CI)** | **OR(95% CI)** |  | **OR(95% CI)** | **OR(95% CI)** |  | **OR(95% CI)** | **OR(95% CI)** |
| **Age, years** |  |  |  |  |  |  |  |  |  |  |  |
| < 55 | 1.00(Ref) | 1.00(Ref) |  | 1.00(Ref) | 1.00(Ref) |  | 1.00(Ref) | 1.00(Ref) |  | 1.00(Ref) | 1.00(Ref) |
| ≥ 55, < 65 | 0.91(0.58-1.42) | 0.81(0.49-1.33) |  | 1.30(0.49-3.47) | 1.24(0.31-4.98) |  | 0.66(0.22-1.93) | 0.03(0.00-0.56) |  | 0.70(0.26-1.89) | 0.41(0.11-1.54) |
| ≥ 65, < 75 | 0.69(0.44-1.10) | 0.79(0.46-1.34) |  | 1.11(0.42-2.95) | 1.25(0.29-5.29) |  | 0.90(0.26-3.14) | 0.00(0.00-0.31) |  | 1.34(0.41-4.34) | 1.41(0.24-8.45) |
| ≥ 75 | 0.61(0.36-1.05) | 0.68(0.36-1.29) |  | 0.40(0.13-1.26) | 0.44(0.08-2.38) |  | 0.20(0.01-2.67) | NA ^b^ |  | 0.61(0.14-2.69) | 0.47(0.04-6.01) |
| **Sex** |  |  |  |  |  |  |  |  |  |  |  |
| Male | 1.00(Ref) | 1.00(Ref) |  | 1.00(Ref) | 1.00(Ref) |  | 1.00(Ref) | 1.00(Ref) |  | 1.00(Ref) | 1.00(Ref) |
| Female | 1.42(0.97-2.09) | 1.74(1.10-2.74) |  | 2.96(1.38-6.38) | 5.47(1.58-18.99) |  | 0.83(0.30-2.35) | 59.12(2.65-1320.94) |  | 0.79(0.32-1.94) | 0.63(0.16-2.49) |
| **Smoking status** |  |  |  |  |  |  |  |  |  |  |  |
| Never | 1.00(Ref) | 1.00(Ref) |  | 1.00(Ref) | 1.00(Ref) |  | 1.00(Ref) | 1.00(Ref) |  | 1.00(Ref) | 1.00(Ref) |
| Former | 0.94(0.64-1.37) | 0.98(0.63-1.51) |  | 1.16(0.60-2.24) | 1.06(0.44-2.57) |  | 0.86(0.28-2.66) | NA |  | 1.15(0.47-2.81) | 1.45(0.37-5.58) |
| Current | 2.52(1.49-4.26) | 2.03(1.12-3.69) |  | 0.61(0.15-2.48) | 0.47(0.07-3.27) |  | 1.95(0.59-6.47) | NA |  | 0.36(0.09-1.51) | 0.14(0.02-1.19) |
| **Etiology** |  |  |  |  |  |  |  |  |  |  |  |
| Alcohol | 1.00(Ref) | 1.00(Ref) |  | 1.00(Ref) | 1.00(Ref) |  | 1.00(Ref) | 1.00(Ref) |  | 1.00(Ref) | 1.00(Ref) |
| HBV + HCV | 1.40(0.85-2.30) | 1.13(0.64-1.98) |  | 1.73(0.71-4.25) | 1.17(0.33-4.15) |  | 1.20(0.16-8.79) | NA |  | 0.92(0.04-19.80) | 0.32(0.00-22.71) |
| HBV + HCV + Alcohol | 2.51(1.56-4.05) | 2.04(1.21-3.45) |  | 3.74(1.39-10.07) | 3.49(1.11-10.95) |  | 0.45(0.07-2.97) | NA |  | 2.41(0.10-58.89) | 1.01(0.01-88.17) |
| NASH | 1.02(0.47-2.20) | 0.86(0.39-1.92) |  | 0.73(0.23-2.33) | 0.50(0.10-2.58) |  | NA | NA |  | NA | NA |
| Other ^c^ | 1.11(0.70-1.77) | 0.96(0.57-1.62) |  | 3.54(1.35-9.30) | 2.70(0.79-9.19) |  | 0.16(0.02-1.42) | NA |  | 0.69(0.02-19.73) | 0.44(0.00-40.82) |
| **Child-Pugh score** |  |  |  |  |  |  |  |  |  |  |  |
| A | 1.00(Ref) | 1.00(Ref) |  | 1.00(Ref) | 1.00(Ref) |  | 1.00(Ref) | 1.00(Ref) |  | 1.00(Ref) | 1.00(Ref) |
| B | 1.47(0.94-2.30) | 1.44(0.88-2.36) |  | 2.68(1.24-5.78) | 2.88(1.11-7.51) |  | 1.07(0.31-3.63) | 1.34(0.13-14.05) |  | 0.43(0.13-1.43) | 0.30(0.07-1.43) |
| C | 1.59(0.58-4.33) | 2.41(0.85-6.86) |  | NA | NA |  | NA | NA |  | 0.83(0.04-17.70) | 0.90(0.01-69.14) |
| **Portal vein thrombosis** |  |  |  |  |  |  |  |  |  |  |  |
| No | 1.00(Ref) | 1.00(Ref) |  | 1.00(Ref) | 1.00(Ref) |  | 1.00(Ref) | 1.00(Ref) |  | 1.00(Ref) | 1.00(Ref) |
| Yes | 1.24(0.86-1.78) | 0.91(0.59-1.41) |  | 3.24(1.39-7.54) | 2.59(0.81-8.30) |  | 1.14(0.41-3.13) | 0.24(0.02-2.40) |  | 0.79(0.32-1.96) | 0.28(0.07-1.17) |
| **NCCN tumor stage** |  |  |  |  |  |  |  |  |  |  |  |
| I | 1.00(Ref) | 1.00(Ref) |  | 1.00(Ref) | 1.00(Ref) |  | 1.00(Ref) | 1.00(Ref) |  | 1.00(Ref) | 1.00(Ref) |
| II | 1.76(0.94-3.30) | 1.82(0.93-3.55) |  | 1.29(0.44-3.85) | 0.94(0.23-3.86) |  | 0.61(0.11-3.34) | 0.00(0.00-0.37) |  | 3.07(0.52-18.24) | 3.81(0.39-36.84) |
| III | 2.09(1.21-3.60) | 2.08(1.14-3.82) |  | 2.90(1.14-7.36) | 2.08(0.64-6.75) |  | 1.32(0.30-5.88) | 0.19(0.01-2.79) |  | 4.56(1.20-17.35) | 14.14(2.09-95.79) |
| IV | 1.74(0.98-3.08) | 1.65(0.86-3.15) |  | 3.37(1.30-8.77) | 2.94(0.88-9.91) |  | 1.27(0.29-5.57) | 0.71(0.06-8.15) |  | 3.67(0.95-14.17) | 13.43(2.08-86.91) |
| **Comorbidity** |  |  |  |  |  |  |  |  |  |  |  |
| No | 1.00(Ref) | 1.00(Ref) |  | 1.00(Ref) | 1.00(Ref) |  | 1.00(Ref) | 1.00(Ref) |  | 1.00(Ref) | 1.00(Ref) |
| 1 | 1.65(0.90-3.02) | 1.88(0.97-3.67) |  | 0.70(0.22-2.22) | 0.92(0.21-4.02) |  | 1.85(0.45-7.54) | 4.46(0.30-67.33) |  | 2.35(0.74-7.47) | 2.41(0.48-12.09) |
| 2 | 1.28(0.72-2.28) | 1.77(0.94-3.34) |  | 0.65(0.23-1.87) | 1.67(0.36-7.66) |  | 0.82(0.21-3.15) | 4.96(0.31-78.50) |  | 3.41(1.10-10.61) | 3.44(0.56-21.29) |
| > 2 | 1.45(0.82-2.56) | 1.99(1.06-3.76) |  | 0.77(0.28-2.13) | 2.01(0.46-8.80) |  | 1.59(0.37-6.86) | 5.68(0.35-91.92) |  | 1.49(0.37-5.99) | 1.06(0.11-10.36) |
| **Prior treatment** |  |  |  |  |  |  |  |  |  |  |  |
| No | 1.00(Ref) | 1.00(Ref) |  | 1.00(Ref) | 1.00(Ref) |  | 1.00(Ref) | 1.00(Ref) |  | 1.00(Ref) | 1.00(Ref) |
| Curative | 0.95(0.55-1.66) | 1.08(0.55-2.09) |  | 0.37(0.06-2.19) | 0.34(0.03-4.41) |  | 0.27(0.08-0.94) | 0.03(0.00-0.73) |  | 1.39(0.31-6.22) | 2.28(0.25-20.54) |
| Palliative | 0.99(0.63-1.55) | 0.82(0.51-1.33) |  | 0.78(0.33-1.84) | 0.43(0.14-1.32) |  | 2.19(0.58-8.34) | NA |  | 0.96(0.28-3.30) | 0.34(0.06-1.99) |
| **CA 19-9 (U/ml)** |  |  |  |  |  |  |  |  |  |  |  |
| ≤ 35 | 1.00(Ref) | 1.00(Ref) |  | 1.00(Ref) | 1.00(Ref) |  | 1.00(Ref) | 1.00(Ref) |  | 1.00(Ref) | 1.00(Ref) |
| > 35, ≤ 100 | 1.05(0.59-1.87) | 1.16(0.60-2.25) |  | 1.59(0.56-4.50) | 3.20(0.46-22.09) |  | 1.50(0.26-8.82) | NA |  | 1.52(0.33-7.01) | NA |
| > 100 | 1.19(0.65-2.16) | 0.98(0.47-2.05) |  | 2.24(0.62-8.07) | 0.81(0.10-6.82) |  | NA | NA |  | 1.14(0.29-4.39) | NA |
| **ALP (U/L)** |  |  |  |  |  |  |  |  |  |  |  |
| ≤ 126 | 1.00(Ref) | 1.00(Ref) |  | 1.00(Ref) | 1.00(Ref) |  | 1.00(Ref) | 1.00(Ref) |  | 1.00(Ref) | 1.00(Ref) |
| > 126, ≤ 200 | 1.25(0.71-2.22) | 1.14(0.59-2.19) |  | 1.23(0.39-3.90) | 0.45(0.08-2.61) |  | 3.85(0.28-52.98) | NA |  | 5.30(0.61-46.23) | NA |
| > 200 | 1.39(0.79-2.44) | 1.34(0.65-2.75) |  | 2.15(0.75-6.14) | 0.73(0.11-4.74) |  | 1.74(0.38-8.07) | NA |  | 5.30(0.74-37.75) | NA |
| **Direct bilirubin (mg/dl)** |  |  |  |  |  |  |  |  |  |  |  |
| ≤ 0.4 | 1.00(Ref) | 1.00(Ref) |  | 1.00(Ref) | 1.00(Ref) |  | 1.00(Ref) | 1.00(Ref) |  | 1.00(Ref) | 1.00(Ref) |
| > 0.4 | 2.42(1.30-4.50) | 3.47(1.54-7.80) |  | 1.84(0.60-5.64) | 0.63(0.06-6.42) |  | 1.90(0.45-8.06) | NA |  | 0.46(0.08-2.68) | NA |
| **Serum albumin (g/dl)** |  |  |  |  |  |  |  |  |  |  |  |
| ≥ 3.5 | 1.00(Ref) | 1.00(Ref) |  | 1.00(Ref) | 1.00(Ref) |  | 1.00(Ref) | 1.00(Ref) |  | 1.00(Ref) | 1.00(Ref) |
| ≥ 3.2, < 3.5 | 1.79(0.93-3.45) | 1.82(0.82-4.01) |  | 0.95(0.30-3.06) | 0.50(0.07-3.63) |  | 2.95(0.46-18.77) | NA |  | NA | NA |
| < 3.2 | 1.79(0.84-3.81) | 1.62(0.56-4.68) |  | 3.01(0.79-11.49) | 3.74(0.27-51.86) |  | 5.15(0.49-54.31) | NA |  | 1.68(0.07-41.89) | NA |
| **WBC (× 10^9^/L)** |  |  |  |  |  |  |  |  |  |  |  |
| 4-11 | 1.00(Ref) | 1.00(Ref) |  | 1.00(Ref) | 1.00(Ref) |  | 1.00(Ref) | 1.00(Ref) |  | 1.00(Ref) | 1.00(Ref) |
| < 4 | 1.18(0.61-2.29) | 0.99(0.45-2.15) |  | 1.37(0.35-5.27) | 0.36(0.02-7.92) |  | 0.84(0.04-16.29) | NA |  | NA | NA |
| > 11 | 1.18(0.48-2.90) | 1.56(0.58-4.22) |  | 5.80(1.07-31.30) | NA |  | NA | NA |  | NA | NA |
| **Lymphocytes (× 10^9^/L)** |  |  |  |  |  |  |  |  |  |  |  |
| ≥ 1.0 | 1.00(Ref) | 1.00(Ref) |  | 1.00(Ref) | 1.00(Ref) |  | 1.00(Ref) | 1.00(Ref) |  | 1.00(Ref) | 1.00(Ref) |
| < 1.0 | 1.62(0.97-2.70) | 1.77(0.98-3.20) |  | 2.17(0.73-6.48) | 0.37(0.06-2.09) |  | 0.37(0.08-1.75) | NA |  | 2.17(0.35-13.38) | NA |
| **Monocytes (× 10^9^/L)** |  |  |  |  |  |  |  |  |  |  |  |
| ≤ 0.7 | 1.00(Ref) | 1.00(Ref) |  | 1.00(Ref) | 1.00(Ref) |  | 1.00(Ref) | 1.00(Ref) |  | 1.00(Ref) | 1.00(Ref) |
| > 0.7 | 0.99(0.60-1.62) | 0.98(0.57-1.68) |  | 3.76(1.14-12.41) | 15.51(1.38-173.81) |  | 2.91(0.69-12.31) | NA |  | 0.26(0.02-3.16) | NA |
| **Neutrophils (× 10^9^/L)** |  |  |  |  |  |  |  |  |  |  |  |
| ≤ 7.3 | 1.00(Ref) | 1.00(Ref) |  | 1.00(Ref) | 1.00(Ref) |  | 1.00(Ref) | 1.00(Ref) |  | 1.00(Ref) | 1.00(Ref) |
| > 7.3 | 1.47(0.74-2.91) | 2.13(0.96-4.72) |  | 5.54(1.04-29.61) | NA |  | 3.11(0.52-18.51) | NA |  | 1.84(0.08-44.93) | NA |
| **NLR** |  |  |  |  |  |  |  |  |  |  |  |
| ≤ 4.0 | 1.00(Ref) | 1.00(Ref) |  | 1.00(Ref) | 1.00(Ref) |  | 1.00(Ref) | 1.00(Ref) |  | 1.00(Ref) | 1.00(Ref) |
| > 4.0 | 1.70(1.04-2.80) | 2.05(1.13-3.73) |  | 1.89(0.68-5.23) | 3.27(0.61-17.59) |  | 1.56(0.39-6.20) | NA |  | 0.66(0.11-3.99) | NA |
| **LMR** |  |  |  |  |  |  |  |  |  |  |  |
| ≤ 2.9 | 1.00(Ref) | 1.00(Ref) |  | 1.00(Ref) | 1.00(Ref) |  | 1.00(Ref) | 1.00(Ref) |  | 1.00(Ref) | 1.00(Ref) |
| > 2.9 | 0.62(0.38-1.02) | 0.60(0.34-1.08) |  | 0.49(0.20-1.20) | 0.30(0.07-1.31) |  | 0.44(0.12-1.70) | NA |  | 1.52(0.31-7.47) | NA |

Abbreviations: ALP, alkaline phosphatase; CA19-9, carbohydrate antigen 19-9; CI, confidence Interval; HBV, hepatitis B virus; HCV, hepatitis C virus; LMR, lymphocyte-to-monocyte ratio; MCS, Mental Component Summary; NASH, non-alcoholic steatohepatitis; NCCN, National Comprehensive Cancer Network; NLR, neutrophil-to-lymphocyte ratio; OR, odds ratio; WBC, white blood cell.

^a^ Adjusted for sex, age, smoking, etiology, Child-Pugh score, cancer stage, portal vein thrombosis, comorbidity, and prior treatment if appropriate.

^b^ Data were not available because of small sample size.

^c^ Including cryptogenic (N = 128), poison (N = 6), autoimmune (N = 5), hemochromatosis (N = 5), primary biliary cirrhosis (N = 1), and estrogen (N = 1).
