# Supplementary material for: Elevated systemic inflammatory responses, factors associated with physical and mental quality of life, and prognosis of hepatocellular carcinoma
Source: Aging (Albany NY). 2020 Mar 7;12(5):4357–70. doi: 10.18632/aging.102889 (PMC7093167; doi:10.18632/aging.102889)
Supplement: Supplementary Table 5 [file aging-12-102889-s001..docx]

**Supplementary Table 5. Association of PCS/MCS score with five-year overall survival (stratified analyses by sex, prior treatment, cirrhosis, and portal vein hypertension history)**

| **Variables** | **PCS ^a^** | | | |  | **MCS ^b^** | | | |
| --- | --- | --- | --- | --- | --- | --- | --- | --- | --- |
|  | **Adjusted HR ^c^ (95% CI)** | ***P* value** | **MST** | **Log rank *P*** |  | **Adjusted HR ^c^ (95% CI)** | ***P* value** | **MST** | **Log rank *P*** |
| **Male** |  |  |  |  |  |  |  |  |  |
| High score | 1.00 (Ref) |  | 21.2 |  |  | 1.00 (Ref) |  | 16.9 |  |
| Medium score | 1.50 (1.17-1.92) | **0.001** | 12.3 |  |  | 0.93 (0.72-1.18) | 0.54 | 15.0 |  |
| Low score | 1.74 (1.33-2.27) | **< 0.001** | 8.1 | **< 0.001** |  | 1.31 (1.02-1.68) | **0.04** | 8.2 | **< 0.001** |
| ***P*  for trend** |  | **< 0.001** |  |  |  |  | 0.05 |  |  |
| **Female** |  |  |  |  |  |  |  |  |  |
| High score | 1.00 (Ref) |  | 26.0 |  |  | 1.00 (Ref) |  | 19.7 |  |
| Medium score | 1.46 (0.89-2.39) | 0.14 | 16.5 |  |  | 1.19 (0.72-1.98) | 0.50 | 18.5 |  |
| Low score | 1.85 (1.02-3.36) | **0.04** | 10.7 | **< 0.001** |  | 0.99 (0.60-1.64) | 0.97 | 14.5 | 0.71 |
| ***P*  for trend** |  | **0.04** |  |  |  |  | 0.92 |  |  |
| **No prior treatment** |  |  |  |  |  |  |  |  |  |
| High score | 1.00 (Ref) |  | 21.8 |  |  | 1.00 (Ref) |  | 15.4 |  |
| Medium score | 1.43 (1.12-1.83) | **0.005** | 12.3 |  |  | 0.88 (0.69-1.13) | 0.33 | 14.1 |  |
| Low score | 1.52 (1.16-1.99) | **0.002** | 6.8 | **< 0.001** |  | 1.18 (0.92-1.51) | 0.19 | 8.1 | **0.005** |
| ***P*  for trend** |  | **0.002** |  |  |  |  | 0.19 |  |  |
| **Prior treatment** |  |  |  |  |  |  |  |  |  |
| High score | 1.00 (Ref) |  | 30.0 |  |  | 1.00 (Ref) |  | 18.5 |  |
| Medium score | 3.08 (1.83-5.20) | **< 0.001** | 16.5 |  |  | 1.45 (0.90-2.35) | 0.13 | 18.9 |  |
| Low score | 2.55 (1.48-4.39) | **< 0.001** | 13.7 | **< 0.001** |  | 1.34 (0.76-2.37) | 0.31 | 14.7 | 0.60 |
| ***P*  for trend** |  | **< 0.001** |  |  |  |  | 0.23 |  |  |
| **No cirrhosis** |  |  |  |  |  |  |  |  |  |
| High score | 1.00 (Ref) |  | 25.7 |  |  | 1.00 (Ref) |  | 19.3 |  |
| Medium score | 1.33 (0.94-1.89) | 0.11 | 12.5 |  |  | 0.80 (0.55-1.16) | 0.23 | 16.3 |  |
| Low score | 1.55 (1.06-2.26) | **0.03** | 8.8 | **< 0.001** |  | 1.12 (0.76-1.65) | 0.56 | 9.4 | **0.03** |
| ***P*  for trend** |  | **0.02** |  |  |  |  | 0.70 |  |  |
| **Cirrhosis** |  |  |  |  |  |  |  |  |  |
| High score | 1.00 (Ref) |  | 20.9 |  |  | 1.00 (Ref) |  | 15.0 |  |
| Medium score | 1.74 (1.30-2.32) | **< 0.001** | 13.8 |  |  | 1.16 (0.88-1.54) | 0.30 | 15.8 |  |
| Low score | 2.14 (1.56-2.94) | **< 0.001** | 8.2 | **< 0.001** |  | 1.31 (0.98-1.76) | 0.07 | 10.7 | 0.14 |
| ***P*  for trend** |  | **< 0.001** |  |  |  |  | 0.07 |  |  |
| **No portal hypertension** | |  |  |  |  |  |  |  |  |
| High score | 1.00 (Ref) |  | 26.0 |  |  | 1.00 (Ref) |  | 19.3 |  |
| Medium score | 1.36 (1.05-1.77) | **0.02** | 15.5 |  |  | 0.89 (0.68-1.15) | 0.38 | 18.5 |  |
| Low score | 1.68 (1.26-2.24) | **< 0.001** | 8.8 | **< 0.001** |  | 1.29 (0.98-1.69) | 0.07 | 10.7 | **0.002** |
| ***P*  for trend** |  | **< 0.001** |  |  |  |  | 0.10 |  |  |
| **Portal hypertension** |  |  |  |  |  |  |  |  |  |
| High score | 1.00 (Ref) |  | 15.6 |  |  | 1.00 (Ref) |  | 10.3 |  |
| Medium score | 2.13 (1.39-3.27) | **< 0.001** | 8.7 |  |  | 1.09 (0.72-1.66) | 0.69 | 12.7 |  |
| Low score | 1.93 (1.23-3.03) | **0.004** | 7.7 | **0.001** |  | 1.35 (0.90-2.03) | 0.14 | 8.0 | 0.99 |
| ***P*  for trend** |  | **0.004** |  |  |  |  | 0.13 |  |  |

Abbreviations: CI, confidence interval; HR, hazard ratio; MCS, Mental Component Summary; MST, median survival time; PCS, Physical Component Summary.

^a^ PCS: High, ≥ 45.0; Medium, ≥ 30.5, < 45.0; Low, < 30.5.

^b^ MCS: High, ≥ 54.4; Medium, ≥ 41.3, < 54.4; Low, < 41.3.

^c^ Adjusted for sex, age, race, BMI, Child-Pugh score, cirrhosis, portal hypertension, portal vein thrombosis, cancer stage, histologic grade, comorbidity, and prior treatment.
